# Interplay of geometrical and spin chiralities in 3D twisted magnetic ribbons

**DOI:** 10.1038/s41586-024-08582-8

**Published:** 2025-02-26

**Authors:** André M. A. Farinha, See-Hun Yang, Jiho Yoon, Banabir Pal, Stuart S. P. Parkin

**Affiliations:** 1 Max Planck Institute of Microstructure Physics, Halle, Germany; 2 Institute of Physics, Martin Luther University, Halle-Wittenberg, Halle, Germany

**Keywords:** Magnetic devices, Spintronics

## Abstract

Chirality is a ubiquitous and fundamental asymmetry in nature^1,2^. Recently, the interaction of chiral objects with spin currents has attracted enormous attention from both scientific and technological perspectives^3–5^. Of particular interest is the current-driven motion of chiral topological excitations such as chiral magnetic domain walls in chiral three-dimensional magnetic structures that could allow for high-density memory-storage devices. Here we use state-of-the-art multiphoton lithography^6,7^ to create three-dimensional chiral magnetic ribbons and perform current-induced motion of chiral domain walls. The ribbons are designed to have a clockwise or anticlockwise chiral twist with a variable magnitude. We find that domain walls can either pass through the ribbon or are impeded, depending on their chirality and configuration and the geometrical chiral twist of the ribbon. The interplay between the magnetic exchange energy and the geometrical twist generates a torsional field that favours chiral Bloch-type walls rather than the Néel-type wall favoured by the intrinsic magnetic properties of the magnetic ribbon itself. Furthermore, the interplay of spin chirality and chiral twist results in a non-reciprocal domain wall motion, namely, a domain wall filter or diode^8–10^. Our findings show how the interplay between geometrical and spin chiralities can lead to new functionalities that could allow for innovative chiral spintronics.

## Abstract (web summary)

The interplay between geometrical and spin chiralities in three-dimensional twisted magnetic ribbons can lead to new functionalities that could allow for innovative chiral spintronics.

## Main

Recent discoveries of means to manipulate chiral domain walls (DWs)^11^ with current, such as spin–orbit torques (SOT) and exchange coupling torques^12,13^, have led to various proposals for magnetic memory^14^, logic^15,16^ and neuromorphic devices^17^. The driving mechanisms are intrinsically associated with chirality that is typically induced by a Dzyaloshinskii–Moriya exchange interaction (DMI) at the interface between an SOT source layer and an adjacent perpendicularly magnetized layer^18^. This DMI gives rise to local chiral fields that stabilize chiral Néel-type walls against the DW shape anisotropy that favours Bloch-type walls and, thereby, drives chiral DWs at the same velocities for both DW configurations ($\odot$⊗ and ⊗$\odot$, where $\odot$ and ⊗ correspond to up and down magnetization, respectively). It has been demonstrated that such a degeneracy can be lifted by geometrical modifications such as curvature^19^ or branching^20^ from two-dimensional (2D) magnetic wires due to DW tilting^21,22^.

By contrast, exchange-induced chirality arising from geometrical modifications such as curvilinear magnetic objects has attracted much attention recently^23–31^. Theoretical studies have shown that geometrical curvature and torsion can induce DMI-like exchange interactions^26,32^. For example, it has been predicted that chiral asymmetry due to this DMI-like effect moves DWs along opposite directions in left- and right-handed magnetic helices^33^, and it has been experimentally shown that these effects can lead to DW auto-motion in spirals^34^. Furthermore, it has been theoretically proposed that such DMI-like effects in curvilinear magnetic systems can be used to stabilize skyrmions that otherwise are hard to obtain from non-curvilinear counterparts^35,36^. However, the experimental confirmation of these effects, and particularly the current-induced manipulation of chiral topological excitations such as chiral DWs in three-dimensional (3D) curvilinear structures, has remained unknown because of the difficulty in the experimental realization of these structures and their electrical characterization. Here we use a new multiphoton lithography (MPL)^37,38^ instrument that we developed based on a modified super-resolution microscope. The system has a voxel size of less than 50 nm and was used to fabricate 3D polymeric scaffolds with very smooth surfaces on which magnetic thin film heterostructures with individual layers just a few angstroms thick are subsequently deposited. Furthermore, we developed a fabrication scheme that made possible direct electrical connections to the devices without affecting the complex 3D structures. Other approaches to fabricating magnetic 3D structures use focused electron-beam-induced deposition (FEBID)^34^ to either directly deposit the magnetic material, making it difficult to deposit the complex thin film heterostructures used here, or to fabricate scaffolds, in which case the fabrication is more complex and likely to affect the final 3D device. By contrast, the higher fabrication rates of MPL and the insulating properties of our scaffolds allow for a simpler fabrication process.

We investigate current-induced DW motion (CIDWM) along 3D twisted magnetic ribbons and find that, unlike straight ribbons, different DW configurations exhibit different velocities and different threshold current densities, resulting, for example, in chiral DW filtering, that is, a DW diode. As shown by our analytical model, the interaction of the geometrical twist with the magnetization in a DW induces an effective geometrical DMI^24–28^, which gives rise to an effective torsional field and a torsional torque that are opposite for opposite DW configurations, contributing to speeding up a DW configuration but slowing down the other in the presence of a chiral spin current. Furthermore, we show that the CIDWM on twisted ribbons matches the predictions of our model in various cases: DWs with different chiralities and spin currents, in the presence of longitudinal fields and in synthetic antiferromagnetic (SAF) films^12,19^.

## 3D twisted magnetic ribbons

**Fig. 1:**
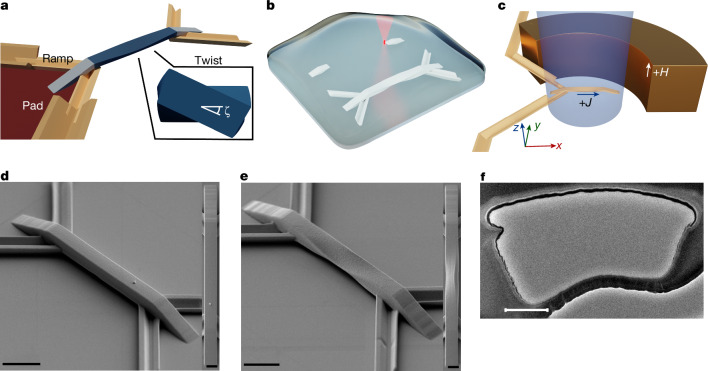
Fabrication and images of 3D magnetic twisted ribbons. **a**, Functional components of the 3D device design. **b**, Schematic of the 3D multiphoton fabrication lithography process. The structure on the right corresponds to the completed device whereas that on the left is under construction using laser scanning exposure. **c**, Schematic of the magneto-optic Kerr effect microscope setup for CIDWM measurements. An out-of-plane magnetic field is used to nucleate a DW. **d**,**e**, SEM perspective and top view of devices with *ζ* = 0° (**d**) and 45° (**e**) twist angles. The total length of the suspended ribbon is 20 μm. In all the SEM images that are shown, a carefully designed protocol has been taken to minimize the exposure of the samples to the electron beam, because we observed that the samples would deform under continuous exposure. **f**, TEM image of a *ζ* = +11° device cross-section. The magnetic film stacks correspond to the black thick convex curve on top of the polymer structure. It is observed that no film is grown on the back side of the structure. Note that the observed undulation cannot be taken as an indication of the surface quality, as the FEBID steps that are necessary to prepare for the TEM acquisition significantly deform the sample surface. For example, all of the accentuated curvature over the top surface is induced by exposure to the electron beam. In this regard, only the thickness of the films coating the polymer core can be reliably determined from the TEM characterization and not the structural properties of the device. Scale bars, 5 μm (**d**,**e**); 500 nm (**f**); 2 μm (**d**, inset; **e**, inset).

The 3D twisted magnetic ribbons were prepared using MPL combined with magnetron sputtering, as shown in Fig. 1a,b,d,e (see the Methods for details). Each device consists of a suspended ribbon, two ramps that provide mechanical support and electrical contacts to the ribbon. A pad definition structure with a V-shaped cross-section is also fabricated that shadows the substrate underneath during the sputter deposition process, thereby preventing a short circuit between the electrical contacts to the device. As a result, the device is ready to measure immediately after film deposition, simplifying the fabrication process. The suspended ribbon is twisted with one chirality in the first half and with the opposite chirality in the second half so that both ends are coplanar. The geometrical twist is characterized by the maximum twist angle *ζ*, measured at the mid-section of the suspended ribbon, and is positive for a right-handed chirality and negative for a left-handed chirality on the left half of the device, as shown in the highlighted regions A and B in Extended Data Fig. 2 and Supplementary Fig. 14. See the Methods for detailed information on the ribbon geometry and Supplementary Notes 4 and 5 for details of the film growth, characterization and CIDWM measurements.

## Current-induced chiral DW motion

**Fig. 2:**
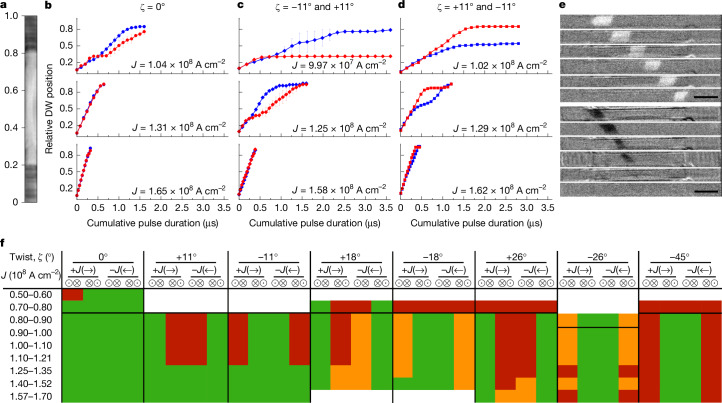
Geometrical twisting effect on the DW motion over the 3D devices. **a**, Top view of a device with a scale of the relative position along the device length. The 3D twisted or suspended section spans from position 0.2 to position 0.8, approximately. **b**–**d**, Relative DW position, as defined in **a**, as a function of the cumulative pulse duration for both DW types moving along +*x* with *ζ* = 0° (**b**), −11° (left half) and +11° (right half) (**c**), +11° (left half) and −11° (right half) (**d**) twisted devices. Red represents $\odot$⊗ and blue represents ⊗$\odot$ DWs, respectively. Circle, diamond and square symbols correspond to *ζ* = 0°, −11° and +11°, and +11° and −11° twisted devices, respectively, as indicated. Each point corresponds to the averaged position from three measurement cycles and the error bars represent the spread in the measurements. **e**, Kerr microscope snapshot images of current-driven DW motion with $\odot$⊗ (top) and ⊗$\odot$ (bottom) configurations on a 3D twisted ribbon with *ζ* = +11° as a function of time. *J* = 1.02 × 10^8^ A cm^−2^ is used. The $\odot$⊗ DW passes through the entire ribbon (top), whereas the ⊗$\odot$ DW is stuck in the middle of the 3D twisted ribbon (bottom). **f**, Qualitative summary of current-driven DW motion along the whole twisted ribbons with different twist angles as a function of *J*. Green corresponds to the case in which the DW can pass through the entire ribbon and red corresponds to the case in which the DW is unable to pass through the entire ribbon. Orange corresponds to the case in which the DW can partially pass through the whole ribbon. Error bars in **b**–**d** represent mean absolute deviations. Scale bar, 5 μm (**e**).

**Extended Data Fig. 1:**
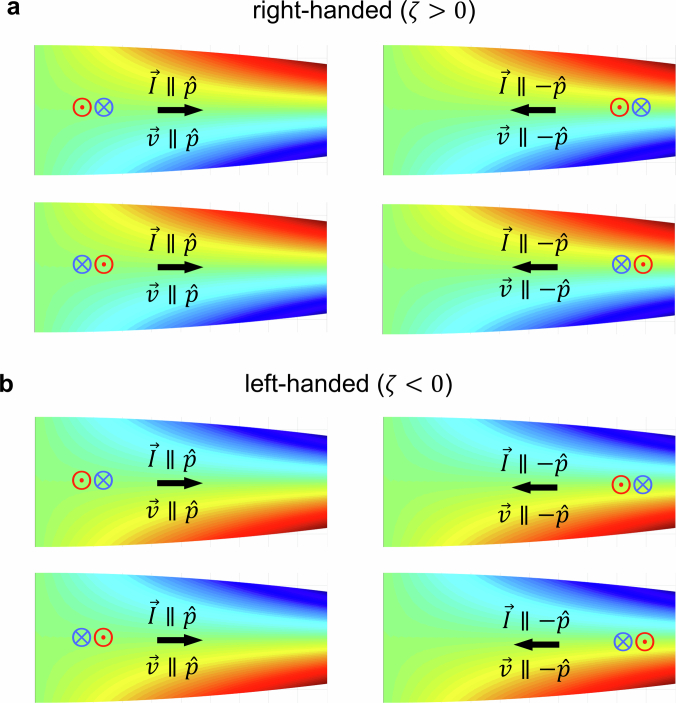
Illustration of chiral twist, domain wall configurations, directions of current and DW motion. **a**, Right-handed chirality of 3D twisted ribbon (the twisting angle *ζ* > 0). **b**, Left-handed chirality of 3D twisted ribbon (*ζ* < 0). **a**,**b**, Current ($\overrightarrow{{\bf{I}}}$) and DW motion ($\overrightarrow{{\bf{v}}}$) directions are parallel with $+\hat{{\bf{p}}}$-direction (left panels). Current and DW motion directions are parallel with $-\hat{{\bf{p}}}$-direction (right panels). See Fig. S3 for the curvilinear coordinates.

First, the DW displacements in the 3D twisted magnetic ribbons (Fig. 2a) are investigated as a function of the cumulative current pulse lengths (Fig. 2b–d). Exemplary Kerr microscope (Fig. 1c) snapshot images of current-driven DW motion in a ribbon with *ζ* = +11° are shown in Fig. 2e (see Extended Data Fig. 1 that shows the chiral twist, the DW configurations and the directions of current and DW motion). Magnetic ribbons formed from a single ferromagnetic (FM) layer with no twists show similar DW displacements for both configurations for all *J*, as expected (Fig. 2b). By contrast, the 3D twisted ribbons show sizeable asymmetries between the two configurations, especially for low *J* (Fig. 2c,d). Most importantly, we find that the asymmetries depend on the magnitude and sign of the chiral twist angle, as shown in Fig. 2f. For low *J* ≈ 1 × 10^8^ A cm^−2^, only one of the DW configurations can pass through the whole ribbon, resulting in a DW filtering, whereas as *J* increases (≳1.25 × 10^8^ A cm^−2^), both DW configurations pass through the ribbon, although at different velocities. We find that the effect of the twisting on the DW motion increases with increasing *ζ* (Fig. 2f). However, more defects are likely to be incorporated into the ribbon with larger *ζ*. This increases *J*_c_ while decreasing the nucleation current density *J*_nuc_, thereby hindering reliable DW motion for |*ζ*| ≥ 18°. Hence, we focus on the devices with *ζ* = ±11°, hereafter.

**Fig. 3:**
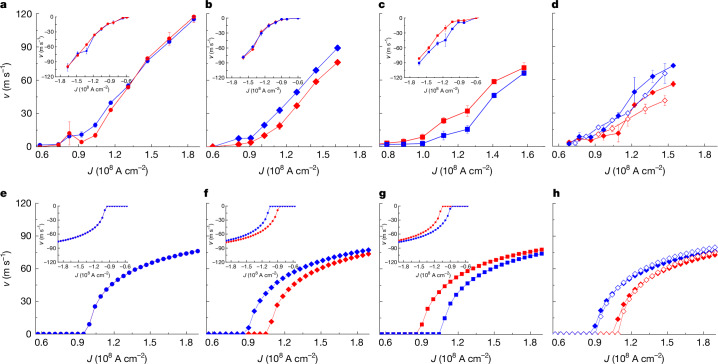
Influence of twisting on the DW velocity in the magnetic ribbons. Red represents the $\odot$⊗ DW configuration and blue represents the ⊗$\odot$ DW configuration. Circle, diamond and square symbols correspond to *ζ* = 0, −11° and +11°, respectively. **a**–**d**, Experimental data. Each point is the average of two to three measurement cycles and the error bars represent the spread in the measurements. Only the CIDWM for the left half of each device is shown. **a**–**c**, *v* for both DW configurations for *J* > 0 for *ζ* = 0 (**a**), −11° (**b**) and +11° (**c**). Inset plots show *v*–*J* for *J* < 0. **d**, *v*–*J* plots for *J* > 0 and *ζ* = −11° with *w* *=* 1.25 μm (solid) and 5 μm (open). **e**–**h**, Corresponding model calculations for the experimental counterparts of **a**–**d**, respectively. The parameters used in the calculations are *t*_p_ = 50 ns, *Δ* = 4 nm, *α* = 0.1, *H*_k_ = 2 kOe, *H*_DM_ = −1 kOe for $\odot$⊗ and 1 kOe for ⊗$\odot$, *M*_s_ = 520 emu cm^−3^, *ξ* = 1 μm^−1^, *A*_ex_ = 3.4 × 10^−6^ erg cm^−1^, *V*_0_ = 8 × 10^5^ erg cm^−3^, *q*_0_ = 40 nm and *q*_shift_ = 0 nm (Supplementary Information Note 2). Error bars in **a**–**d** represent mean absolute deviations.

To investigate these phenomena quantitatively, we focus on CIDWM on the first half of the 3D twisted ribbon. The DW velocities for both $\odot$⊗ and ⊗$\odot$ configurations are shown as a function of current density *J* in Fig. 3. Results are compared for twist angles *ζ* = 0° (no twist) and +11° and −11°. For the straight ribbons, the DW velocities for both configurations are nearly the same for any current polarity (Fig. 3a), as shown in the DW displacements in Fig. 2b. By contrast, for the 3D twisted ribbons, the $\odot$⊗ DW moves faster than the ⊗$\odot$ DW within the +11° twisted region for positive current (Fig. 3c), whereas the velocity of ⊗$\odot$ DW is higher than $\odot$⊗ DW within the −11° twisted region (Fig. 3b). The opposite is true for the negative current polarity, as shown in Fig. 3b,c (insets). Moreover, the faster DW shows lower *J*_c_ (about 8 × 10^7^ A cm^−2^) than the slower counterpart (about 1 × 10^8^ A cm^−2^). Thus, the ⊗$\odot$ DW is filtered out when 8 × 10^7^ A cm^−2^ < *J* < 1 × 10^8^ A cm^−2^ for the region with the +11° twisting angle, whereas the $\odot$⊗ DW is filtered out for the opposite current polarity.

## Torsional fields and torsional torques

**Fig. 4:**
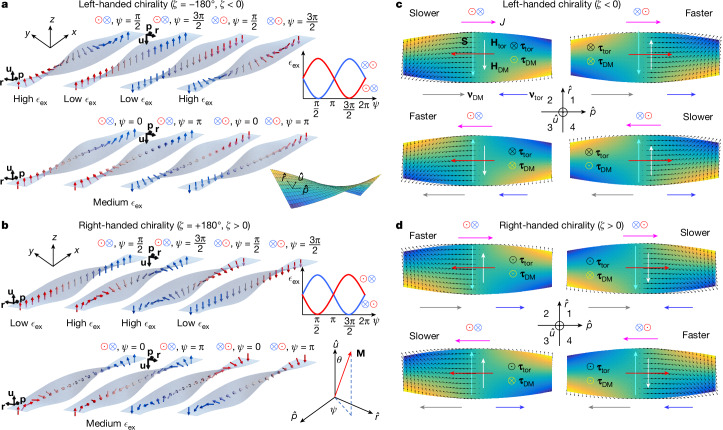
Mechanism of chiral-twist-dependent CIDWM in 3D twisted magnetic ribbons. **a**,**b**, *ϵ*_ex_ as a function of *ψ* = 0, π (Néel-type DW, bottom) and π/2, 3π/2 (Bloch-type DW, top) for two DW configurations with negative (left-handed) (**a**) and positive (right-handed) (**b**) *ζ* in the 3D twisted magnetic ribbon. The arrows correspond to the local DW magnetization. Note here that the DW width equals the twisting length to illustrate how *ϵ*_ex_ depends on *ϕ* and *ζ*. $(\hat{{\bf{p}}},\hat{{\bf{r}}},\hat{{\bf{u}}})$ are the orthogonal unit vectors, respectively, on the ribbon surface. *θ* and *ψ* correspond to the polar and azimuthal angles of the DW magnetization, respectively, in the $(\hat{{\bf{p}}},\hat{{\bf{r}}},\hat{{\bf{u}}})$ coordinate (Supplementary Fig. 3a). **c**,**d**, Illustration of various chiralities and torques that are associated with CIDWM in the 3D twisted magnetic ribbons for two DW configurations with negative (left-handed) (**c**) and positive (right-handed) *ζ* (**d**).

Let us discuss the underlying mechanism of how the chiral twist affects the current-driven motion of DWs. It is assumed that (1) the DW width (π*Δ* where *Δ* is the DW width parameter), estimated to be about 12 nm for our devices, is much smaller than the ribbon width; (2) the DW tilting angle *χ* is small; and (3) *J* is not large (≲2 × 10^8^ A cm^−2^ in our devices). *χ* is the angle between the DW direction and the transverse direction to the ribbon (Supplementary Fig. 4), for example, *χ* = 0 when a DW is untilted and transverse to the ribbon. Note that the polar angle *θ* of the DW magnetization changes from 0 to π for the $\odot$⊗ DW configuration and from π to 0 for the ⊗$\odot$ DW configuration for perpendicularly magnetized domains within the curvilinear coordinate system in the 3D twisted ribbon, as shown in Fig. 4a. $(\hat{{\bf{p}}},\hat{{\bf{r}}},\hat{{\bf{u}}})$ is a set of mutually orthogonal unit vectors at the ribbon surface (Fig. 4a). By contrast, the azimuthal angle *ψ* of the magnetization not only determines the type of DW, that is, *ψ* = 0, π (Néel) and π/2, 3π/2 (Bloch) but strongly affects the local magnetic exchange energy per unit volume, *ϵ*_ex_, depending on the chiral twist (Fig. 4a,b). Note that the larger (steeper) magnetization gradients within the DWs give rise to larger *ϵ*_ex_, as ${{\epsilon }}_{{\rm{e}}{\rm{x}}}={A}_{{\rm{e}}{\rm{x}}}{|{\rm{\nabla }}\hat{{\bf{m}}}|}^{2}$, where *A*_ex_ is the exchange stiffness and $\hat{{\bf{m}}}=\frac{{\bf{M}}}{|{\bf{M}}|}$ (the magnetization unit vector). For the Néel-type wall case, *ϵ*_ex_ (*ψ* = 0) = *ϵ*_ex_ (*ψ* = π), corresponding to a medium value of *ϵ*_ex_, as shown in Fig. 4a,b.

By contrast, *ϵ*_ex_ forms a minimum and a maximum for the Bloch-type DWs depending on the DW configuration and chiral twist (Fig. 4a,b). For example, *ϵ*_ex_ is the lowest at *ψ* *=* π/2 for (that is, $\odot$ ↑ $\otimes$) for a right-handed chiral twist (*ζ* > 0). In this case, the rotation of $\hat{{\bf{m}}}$ in the DW is partially compensated by a twisting of the ribbon in the opposite direction, in such a way that the magnetization gradient ${\rm{\nabla }}\hat{{\bf{m}}}$, and, therefore, *ϵ*_ex_ is smaller. By contrast, *ϵ*_ex_ is the largest at *ψ* *=* 3π/2 for the same configuration (↓) and *ζ* > 0, because the DW magnetization rotates in the same direction as the ribbon twists around the $\hat{{\bf{p}}}$ direction. Thus, this gives rise to a steep rotation and large gradient of $\hat{{\bf{m}}}$ within the DW. This difference in *ϵ*_ex_ between *ψ* *=* π/2 (minimum *ϵ*_ex_) and *ψ* *=* 3π/2 (maximum *ϵ*_ex_) generates an effective torsional field ${{\bf{H}}}_{{\rm{D}}{\rm{M}}}=-\frac{\delta {{\epsilon }}_{{\rm{e}}{\rm{x}}}}{\delta {\bf{M}}}=\frac{2\xi {A}_{{\rm{e}}{\rm{x}}}}{{M}_{{\rm{s}}}\varDelta }\hat{{\bf{r}}}$ that is along *ϕ* = π/2 direction (↑) for the right-handed chiral twist, as shown in Fig. 4d. Here *ξ* is the twist parameter defined as *ζ*/*L* (see model description in Supplementary Information Note 2), and *M*_s_ is the saturation magnetization. As for the ⊗$\odot$ DW, *ϵ*_ex_ forms a maximum at *ψ* = π/2 and a minimum at *ψ* = 3π/2 such that the difference in *ϵ*_ex_ changes sign, thereby leading to **H**_tor_ along the *ψ* = 3π/2 direction (↓). When the chiral twist is changed, the sign of *ξ* switches, the maximum and minimum of *ϵ*_ex_ are swapped, and, consequently, the orientations of **H**_tor_ are reversed (Fig. 4a). Importantly, note that *ψ* = π/2 and *ψ* = 3π/2 correspond to the left- and right-handed chiral magnetic helices in Bloch walls, respectively. The torsional fields lift the degeneracy of the Bloch walls and favour a certain chirality of the chiral Bloch walls depending on the DW configurations and the chiral twist.

## Interplay of geometrical twisting with spin chiralities

Note that **H**_tor_ induces unidirectionality because of the chiral twist by breaking uniaxial symmetry, thereby favouring a chiral Bloch-type wall. Consequently, **H**_tor_ gives rise to a torsional torque, **τ**_tor_ = −*γ*_tor_**M** × **H**_tor_, acting on **M**, where *γ* is the gyromagnetic ratio. If the volume spin-transfer torque and the anisotropy field torque due to the shape anisotropy of DW are neglected, the total DW velocity is determined by **τ**_tor_ + **τ**_DM_. Note that **τ**_DM_ = −*γ***M** × **H**_DM_ is the DMI torque, the main driving torque to move the chiral DWs, arising from the interface DMI field ${{\bf{H}}}_{{\rm{D}}{\rm{M}}}=-\frac{\delta {{\epsilon }}_{{\rm{D}}{\rm{M}}}}{\delta {\bf{M}}}=-\frac{D}{{M}_{{\rm{s}}}\varDelta }\hat{{\bf{p}}}$. **H**_DM_ favours a Néel-type wall with the left-handed chirality in the Pt/Co system^18^. Here *ϵ*_DM_ and *D* are the DMI energy per unit volume and DMI constant (DMI energy per unit area), respectively. Hence, the DW is neither purely Néel-type nor Bloch-type in the absence of current such that *ψ* is determined by **H**_tor_ + **H**_DM_, that is, $\psi =\arctan \frac{{H}_{{\rm{tor}}}}{{H}_{{\rm{DM}}}}$ (here the DW shape anisotropy field is neglected, for simplicity). For example, *ψ* ≈ π/12 using parameters that fit the experimental CIDWM data for *ζ* = ±11° shown in Fig. 3b,c. The experimental results qualitatively agree with our analytical model (see Fig. 3f–h and Extended Data Fig. 3 for time-resolved evolution of the CIDWM). In the configuration being addressed, on application of charge current along ${\bf{J}}\,\parallel +\,\hat{{\bf{p}}}$, the spin Hall effect (SHE) in the Pt underlayer gives rise to a spin current that flows into the Co overlayer, that is ${{\bf{J}}}_{{\rm{s}}}\parallel +\,\hat{{\bf{u}}}$. The direction of the spin-polarization^39^, ${\bf{S}}\,\parallel \pm \hat{{\bf{r}}}$, is determined by the sign of the spin Hall parameter (${H}_{{\rm{SH}}}=\frac{\hbar {\theta }_{{\rm{SH}}}\,J}{2eM{t}_{{\rm{FM}}}}$, where *θ*_SH_ is the spin Hall angle and *t*_FM_ is the ferromagnetic layer thickness), which, in the case of Pt, leads to ${\bf{S}}\,\parallel -\,\hat{{\bf{r}}}$ (Extended Data Table 1). This chiral spin current exerts a damping-like SOT on **M**, thereby rotating **M** towards **S** and, consequently, placing **M** in a specific quadrant of the plane defined by $\hat{{\bf{p}}}$ and $\hat{{\bf{r}}}$ (Fig. 4a,c,d (insets)). For example, when **M** is in the third quadrant that corresponds to the case and ${{\bf{H}}}_{{\rm{D}}{\rm{M}}}\,\parallel -\,\hat{{\bf{p}}}$ for the $\odot$⊗ DW (Fig. 4c,d, top left), *τ*_DM_ is along $+\hat{{\bf{u}}}$. At the same time, the chiral twist with *ζ* > 0 for the $\odot$⊗ DW induces ${{\bf{H}}}_{{\rm{t}}{\rm{o}}{\rm{r}}}\parallel +\hat{{\bf{r}}}$, and thus *τ*_tor_ is generated along $+\hat{{\bf{u}}}$ (Fig. 4d, left). Consequently, the total torque ${{\boldsymbol{\tau }}}_{{\rm{t}}{\rm{o}}{\rm{t}}}={{\boldsymbol{\tau }}}_{{\rm{t}}{\rm{o}}{\rm{r}}}(\,+\hat{{\bf{u}}}:\odot \,)+{{\boldsymbol{\tau }}}_{{\rm{D}}{\rm{M}}}(\,+\hat{{\bf{u}}}:\odot \,)$ moves the $\odot$⊗ DW faster (Fig. 4d, top left). By contrast, when the chiral twist switches such that *ζ* < 0, ${{\bf{H}}}_{{\rm{t}}{\rm{o}}{\rm{r}}}\,\parallel -\,\hat{{\bf{r}}}$ and *τ*_tor_ is induced along $-\hat{{\bf{u}}}$ so that ${{\boldsymbol{\tau }}}_{{\rm{t}}{\rm{o}}{\rm{t}}}={{\boldsymbol{\tau }}}_{{\rm{t}}{\rm{o}}{\rm{r}}}(\,+\hat{{\bf{u}}}:\odot \,)+{{\boldsymbol{\tau }}}_{{\rm{D}}{\rm{M}}}(\,-\hat{{\bf{u}}}:\otimes \,)$ moves $\odot$⊗ DW slowly (Fig. 4c, top left). The other cases can be readily accounted for by this mechanism, as shown in Fig. 4c,d. Note that **τ**_tor_ always points along $+\hat{{\bf{u}}}$ ($\odot$) for *ζ* > 0, whereas it is aligned along $-\hat{{\bf{u}}}$ (⊗) for *ζ* < 0, irrespective of the DW configuration and current direction (see ${{\boldsymbol{\tau }}}_{{\rm{t}}{\rm{o}}{\rm{r}}}^{r}$ in Extended Data Table 1). This shows that the asymmetry induced by *τ*_tor_ is distinct from *τ*_DM_, the direction of which switches as the DW configurations and current directions are reversed.

Next let us investigate the chiral geometrical twisting effect as a function of *J* and ribbon width *w*. As the SOT, that is proportional to *J*, brings **M** closer to *ψ* = π/2 or 3π/2 (Bloch-type wall) with increasing *J*, *τ*_tor_ and *v*_tor_ decrease with increasing *J* (*v*_tor_ is the twist-induced DW velocity). This is distinct from *τ*_DM_ and *v*_DM_ that monotonically increase but saturate with increasing *J*. Note that, at *ψ* = π/2 or 3π/2, *τ*_tor_ is zero, whereas *τ*_DM_ forms a maximum. Consequently, δ*v*_tor_ = *v*_tor_ (*ζ* > 0) − *v*_tor_ (*ζ* < 0) decreases with increasing *J* such that δ*v*_tor_ becomes significant at low *J*. This results in a sizeable difference in *J*_c_, as shown in our experimental observations and model. By contrast, we find that the *w*-dependence of δ*v*_tor_ is small for a given *ζ*, as shown in Fig. 3d,h, as *τ*_tor_ is independent of *w* (Supplementary Information Note 2). This is distinct from 2D curved wires in which the velocity difference increases with *w*/*R*, where *R* is the radius of curvature^19^. Moreover, *v*_tor_ is independent of *Δ* (Supplementary Information Note 2), which is similar to the volume spin-transfer torque-driven velocity *v*_STT_ but distinct from *v*_DM_ that is proportional to *Δ* (ref. ^42^). This is because *E*_ex_ increases with decreasing *Δ* for a given *ζ*, thereby leading to *H*_tor_ ∝ 1/*Δ* and *τ*_tor_ ∝ 1/*Δ* (see Supplementary Information Note 2). Note that the DW displacement and velocity with a given torque is proportional to *Δ*, that is, *v*_tor_ ∝ *τ*_tor_*Δ* such that *v*_tor_ is constant with respect to *Δ*.

The longitudinal field *H*_*x*_ dependence of *v* provides additional insight into the interaction of the different chiralities as *H*_*x*_ is parallel to *H*_DM_, effectively adding to or subtracting from *H*_DM_ (ref. ^18^). The *v*–*H*_*x*_ curves are nearly symmetric for the magnetic ribbons with no twisting. By contrast, there are marked asymmetries with respect to not only *J* but also *H*_*x*_ in the twisted ribbons, as shown in Extended Data Fig. 8a–d. The asymmetry in *v* increases with increasing |*H*_*x*_| as *H*_*x*_ brings **M** to *ψ* = 0 or π (Néel-type wall) and *τ*_tor_ increases for a given SOT (Extended Data Fig. 8a–d). For example, for the ⊗$\odot$ DW configuration with *ζ* = −11°, a positive *H*_*x*_ brings the DW magnetization closer to *ψ* = 0 thus increasing the magnitude of *v*_tor_ > 0 that is added to *v*_DM_ in the presence of *J* > 0; for the $\odot$⊗ DW configuration with *ζ* = −11°, a negative *H*_*x*_ brings the DW magnetization closer to *ψ* = π thus increasing the magnitude of *v*_tor_ < 0 that is subtracted from *v*_DM_ in the presence of *J * >  0. By contrast, when *J* < 0, *v*_DM_ changes sign from positive to negative, whereas the sign of *v*_tor_ does not change, so that *v*_tor_ > 0 is subtracted from *v*_DM_ for the ⊗$\odot$ configuration and *v*_tor_ < 0 is added to *v*_DM_ <0 for the $\odot$⊗ configuration.

Finally, CIDWM is investigated in SAF 3D twisted ribbons. It is observed that the geometrical twisting effect vanishes in these ribbons, as shown in Extended Data Fig. 8e. This can be accounted for by the fact that *τ*_tor_ on the component DW in the upper sublayer compensates that in the lower sublayer, as the DW configurations for both sublayers are opposite to each other. This is similar to the case of 2D curved wires^19^, thus showing that the SAF can potentially be useful in 3D racetrack devices that require reliable lock-step motion of multiple DWs.

## Conclusions

In summary, we demonstrate the fabrication of 3D twisted magnetic ribbons using an advanced multiphoton lithography setup. There is tremendous interest today in devising truly 3D devices that go beyond the present-day charge-based devices. Here we show how the interplay between geometrical and spin chirality leads to new physics that influences the current-induced motion of chiral DWs in 3D structured conduits. This has much potential for, in particular, 3D racetrack memory devices: Racetrack Memory is a unique device because of its special property of encoding information in domains and moving DWs, that is, the stored data, by torques from spin currents to and through along the magnetic conduits or racetracks. To date, these racetracks have been mostly 2D but our results on geometrical chirality show much potential for 3D racetrack devices, such as process-in-3D racetrack memory and reconfigurable 3D memory and logic devices.

## Methods

### Multiphoton lithography

To fabricate the 3D twisted ribbons, a super-resolution STED microscope (Abberior Instruments) was adapted extensively in both hardware and software, to perform MPL. A functional diagram of the system is shown in Supplementary Fig. 1. A femtosecond Er fibre laser (Menlo Systems C-Fiber 780) is used to generate multiphoton excitation at an 80-MHz repetition rate and at a wavelength of 780 nm. The optical pulse power is controlled by an acousto-optic modulator before focusing the beam onto a liquid photoresist through a Nikon Plan Apo Lambda 100×/1.45 Oil objective lens. Precise motions of the objective along the optical axis (*z*-axis) are controlled by the combination of a motorized focus stage embedded in the microscope (Nikon Ti-2, working distance: 10 mm) and an XYZ piezostage (Physik Instrumente P-545.3C8S PInano, working distances: 200 μm for all axes). XY-motions are achieved by the combination of an XYZ piezostage and a coarse motion stage (Physik Instrumente U-780 PILine XY-stage: working distances: 135 mm (*x*-axis) and 85 mm (*y*-axis)). The coarse motion stage allows for the fabrication of large structures such as contact pads.

The fabrication process is as follows: first, one drop of photoresist is applied onto a borosilicate glass coverslip that is placed on the microscope sample stage. The glass–photoresist interface is automatically positioned by monitoring the reflection from the substrate surface using a photodiode. The interface positioning errors are less than 200 nm. To improve adhesion, the fabrication is started by focusing the laser inside the substrate. During the fabrication, the laser is scanned along the *xy*-axes using a set of galvano scanners (maximum scanning range of 80 μm) and along the *z*-axis using an XYZ piezostage. The photoresist used for fabrication is composed of 7-diethylamino-3-thenoyl-coumarin (DETC, Luxottica Exciton) as the photoinitiator, 0.25 wt% concentration, and pentaerythritol triacrylate (PETA, Merck Sigma-Aldrich) as the monomer. The photoresist is prepared by precisely weighing each component chemical and mixing them with a magnetic stirrer for 20–40 min at room temperature until the photoinitiator is observed to be completely dissolved in the monomer. This type of photoresist has been widely used and characterized in previous experiments with STED-MPL^37^. Laser excitation pulses with 0.2 nJ energy are applied at an 80 MHz repetition rate. The laser is XY-scanned at a speed of 6.25 mm s^−1^ with the galvano scanner. To reduce the surface roughness, the scanning step interval is set to be 25 nm along the *x-* and *z*-axes. After the illumination, the unpolymerized photoresist is removed by submerging the sample in propylene glycol monomethyl ether acetate (PEGMEA, Merck Sigma-Aldrich) for 25 min, followed by submersion in 2-propanol for 5 min. Finally, the sample is carefully dried with dust-free compressed air to avoid residue or dust on the surface.

### Film growth 

After the MPL process is completed, the glass substrate is cut into smaller pieces in preparation for deposition. The magnetic film stacks are deposited onto both the polymer structures and glass substrate by magnetron sputtering at an Ar pressure of 10^−3^ torr at room temperature. The deposition system has a base pressure better than 10^−9^ Torr. The film growth rates are about 0.9 Å s^−1^ for Pt and about 0.2 Å s^−1^ for FM and Ru films. A 100 Å thick TaN layer is first deposited to improve the adhesion and the smoothness of the following films. TaN capping layers, 30 Å and 60 Å thick, are used to prevent the oxidation of the magnetic layers for the SAF and FM films, respectively. Full film stacks are 100 TaN/30 Pt/3 Co/7 Ni/3 Co/60 TaN and 100 TaN/30 Pt/3 Co/7 Ni/1.5 Co/9.5 Ru/3.5 Co/7 Ni/3 Co/30 TaN for FM and SAF films, respectively (all thicknesses are in Å). Although the sputtered beams are not highly directional or collimated, no films are deposited on the substrate surfaces that are shadowed by the suspended sections of the fabricated structure. By contrast, as the substrate is rotated during deposition, the grown films are mostly uniform over the flat and twisted regions in the ribbons.

### Atomic force microscopy

An Asylum Research Cypher atomic force microscope is used to check the roughness of the deposited films. The suspended region of the 3D device with a +11° twist together with a contiguous section of the substrate is scanned along the ribbon direction in tapping (a.c.) mode to minimize the perturbations at the ribbon edges. The pixel size was set to 10 nm with a scan rate of 0.3 Hz and a voltage of 600 mV. The values given in the paper correspond to the root mean square of roughness, *R*_q_. The measured *R*_q_ is about 0.37 nm in the twisted region with *ζ* = +11° (as compared with the approximately 0.1 nm RMS roughness for the substrate itself) (Supplementary Note 4).

### Kerr microscopy

Current-induced DW motion on the 3D twisted magnetic ribbon structures is measured using polar Kerr microscopy at room temperature (Fig. 1c and Supplementary Fig. 2). The polarizer in the Kerr microscope linearly polarizes the incident light. By contrast, a *λ*/4 waveplate is combined with an analyser to measure the Kerr ellipticity, allowing the magnetization contrast along the *z*-axis to be detected. Devices are wire-bonded by contact pads. The device resistances are measured before and after characterization to probe for notable changes in the devices such as deformations from Joule heating, with variations typically less than 1%. A nanosecond pulse generator is used to move the DWs. The rise and fall times of the voltage pulses are 0.3 ns and 0.8 ns, respectively. Pulse lengths longer than 10 ns are typically applied unless DW nucleation is observed. Sometimes 8 ns long pulses are used when higher voltage pulses are needed for the experiment and 10 ns pulses are observed to promote nucleation (owing to heating effects).

First, a DW is nucleated at either end of the ribbon by fields or current pulses, or a combination of both. Then the DW is driven into the twisted region by sequences of current pulses. Differential mode Kerr microscopy is used: an initial image is used as the background that is subtracted from the image being acquired after a sequence of current pulses has been applied. This process significantly improves the magnetic contrast and resolution of images by minimizing the effect of drift and vibrations on the Kerr contrast. Consequently, the processed Kerr image shows two boundaries in the ribbon that correspond to the previous and present DW positions. These complete process cycles (DW nucleation and motion) are automatized and repeated three to five times to check the reproducibility and for statistics. The current pulse length is limited to avoid Joule heating of the ribbons that are poorly thermally connected to the surface.

The upper bound of *J* is limited by DW nucleation, whereas the lower bound corresponds to *J*_c_ below which the speed of the DW is less than 5 m s^−1^. In each measurement cycle, a DW is attempted to be driven along the whole ribbon by applying a sequence of current pulses. Each Kerr image is acquired after the application of current pulses, corresponding to the DW displacement during the pulses (Fig. 2). A sequence of current pulses with a minimum pulse length *t*_p_ = 10 ns is typically applied between each Kerr image acquisition. Pulses that are 7 ns and 30–50 ns long are used for higher and lower *J*, respectively, as the larger *t*_p_ often heats the suspended 3D structures that are rather poorly thermally connected to the substrate. This also limits the maximum *J*, by more than 40% compared with the counterparts on Si substrates, that can be applied and, thereby, the maximum DW velocity. A 10 ns long pulse is typically used, although 7 ns and 30–50 ns long pulses are used for higher and lower *J*, respectively. Each cycle is repeated thrice to check reproducibility. As for the magnetic ribbon with *ζ* = −45°, the sample is placed on a wedge at 22.5° angle to enhance the Kerr image contrast.

### Data processing

A semi-automatic classification algorithm is developed to locate DW positions from the acquired images. First, a denoising process is applied to each image during the acquisition cycle by image reconstruction using a multistage progressive image restoration^38^. After denoising, the ribbon edges are automatically detected, after which the reconstructed images are normalized, and the contours of the magnetization shade are detected and filtered based on the area and aspect ratio. The resulting contour candidates are presented to the user for selection. The DW positions are determined from the boundaries of selected contours, from which the velocity is calculated based on the input parameters, such as pulse lengths. The average velocity along the ribbon is obtained from the initial and final positions of the DWs for each measurement cycle and the corresponding number of applied pulses.

### Vibrating sample magnetometry

The magnetic properties of unpatterned films are characterized by measuring the easy-axis (out-of-plane) and hard-axis (in-plane) loops with a vibrating sample magnetometer (Lakeshore VSM 8600) at room temperature. The applied field range is ±20 kOe (easy axis for SAF films and hard axis) and ±2 kOe (easy axis for FM films). Both FM and SAF films show excellent perpendicular magnetic anisotropy. The SAF films exhibit large AF exchange coupling and a small net magnetization (see Supplementary Note 5 for magnetic properties, including magnetic hysteresis loops, magnetizations, anisotropies and symmetric and antisymmetric exchange interactions).

### Cross-sectional transmission electron microscopy

The uniformity of the film deposition through a cross-section of a device was characterized for a device with *ζ* = −11° through transmission electron microscopy (TEM). The TEM sample preparation was performed using a TESCAN GAIA3 FIB-SEM workstation. First, the suspended sections of the devices were coated with protective layers of C and Pt using focused electron and ion (Ga) beam-induced deposition, Then, a small slice of the device cross-section in the middle of the suspended section was cut for each device, attached to a Cu TEM-grid and gradually polished with decreasing beam current. A final, low-energy polishing step was performed to clean the exposed surfaces from implanted Ga ions. During the electron-beam-induced deposition, the suspended section of the device was seen to deform, leading to the surface curvature and indentation noticeable on the left side of Fig. 1f, but the deformation did not influence the characterization outcome as the film thickness over the polymer scaffold was unaffected and the sliced cross-sections were not further modified during the remainder of the process. High-resolution TEM characterization of the processed cross-section was performed using a JEOL JEM-F200 microscope, at 200 kV. Cross-sectional TEM on the region with *ζ* = +11° shows that the deposited films are uniform and conformally grow on the scaffold surface except near the edges of the ribbon in which the films are thinner with a thickness gradient (Fig. 1f). No film was found to be deposited onto the shadowed areas or the backside of the suspended ribbon. The thinner regions at the ribbon edges may cause DW pinning during current-induced motion.

### 3D twisted magnetic ribbons with different chiral twists

A 3D device is composed of three sections: two ramps, a suspended ribbon, electrical contact leads and a pad delimitation structure (Fig. 1a,b). The ramps not only support the suspended ribbon but also allow for electrical access from contact pads to the suspended ribbon. The suspended ribbon has a twisted region along the longitudinal direction with two different chiral twists at opposite ends of the ribbon so that both ends are horizontal with respect to the ground plane. For example, one half of the twisted region has a left-handed geometrical chirality (−), whereas the other half has a right-handed geometrical chirality (+). V-shaped structures with a 90° cross-section angle are prepared by MPL to electrically isolate the two electrical contact leads to the device. These structures are used to eliminate additional processing that would otherwise be needed. Suspended ribbons were fabricated that are 20 μm long and 1.25 μm, 2.5 μm and 5 μm wide, with *ζ* = 0, ±11°, ±18°, ±26° and ±45°. The 2.5-μm wide ribbons were chosen for detailed measurements. Each ramp has a 10-μm projected length on the ground plane with an elevation angle of 18° for all devices. We define the section within each half of the ribbon that has a constant twist parameter as having an effective twisting length *L* ≈ 4 μm. The twist parameter *ξ* is defined as *ξ* = *ζ*/*L* such that the twisting angle *X* along the twisting axis *x*, is given by *X* = *ξx* (see Supplementary Note 2 for more details).

**Extended Data Fig. 2:**
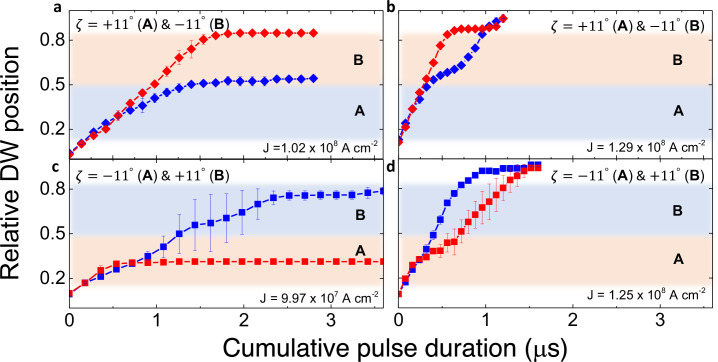
Current-induced DW displacements vs. accumulated current pulse lengths over the entire twisted ribbons having two opposite chiral twist. Two plots are extracted from Fig. 1b with annotations. **a,b**, *ζ* = +11° (left-half: region A) & −11° (right-half: region B) **c**,**d**, *ζ* = −11° (left-half: region A) & +11° (right-half: region B). Region A and B highlight the left and right halves of each ribbon, respectively, that are oppositely twisted to each other. Blue and red regions correspond to right-handed (*ζ* = +11°) and left-handed (*ζ* = −11°) twisted regions, respectively. Red and blue symbols correspond to the $\odot$⊗ and ⊗$\odot$ DW configurations, respectively. Note that tangential slopes of the curves correspond to the respective DW velocities. Error bars represent the average deviation from the mean.

**Extended Data Fig. 3:**
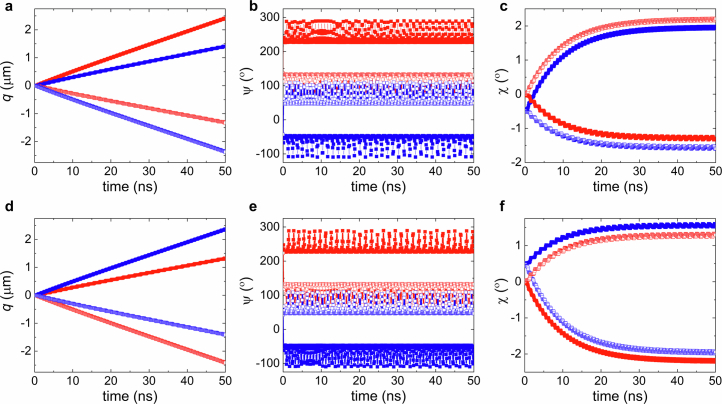
Calculated time-resolved evolution of key parameters in CIDWM. **a**,**d**, DW displacements $q$, **b**,**e**, azimuthal angles of DW magnetization *ψ*. **c**,**f**, DW tilting angles $\chi$. **a**,**b**,**c**, *ζ* = +11° (right-handed). **d**,**e**,**f**, *ζ* = −11° (left-handed). Red and blue symbols correspond to $\odot$⊗ and ⊗$\odot$ configurations, respectively. Solid and open symbols correspond to $I > 0$ and $I < 0$, respectively. The parameters used in the calculations are ${t}_{p}=50$ ns, $J=1.13$ A cm^−2^ (${H}_{{SH}}=0.72$ kOe), $\varDelta =4$ nm, $\alpha =0.1$, ${H}_{k}=2$ kOe, ${H}_{{DM}}=-\,1$ kOe for $\odot$⊗ and 1 kOe for ⊗$\odot$, ${M}_{s}=520$ emu cm^−3^, $L=1$ μm ($\xi =0.19$ μm^−1^), ${A}_{{ex}}=3.4\times {10}^{-6}$ erg cm^−1^, ${V}_{0}=8\times {10}^{5}$ erg cm^−3^, ${q}_{0}=40$ nm, and ${q}_{{shift}}=0$ nm.

The fabricated twisted ribbons have two distinct regions: the ribbon is twisted over one half of its length with one-handedness (region A) and then twisted with the opposite-handedness over the second half (region B), as shown in Extended Data Fig. 2. This enables the ramp structures to be identical at either end of the ribbon (Methods). To investigate the current-driven DW motion in the 3D twisted ribbons with two opposite chiralities, the DW displacements for low and intermediate *J* in Fig. 2c,d are replotted in Extended Data Fig. 2. The data show that the twisting of the ribbon significantly influences the DW motion and strongly depends on the DW configuration, whether $\odot$⊗ or ⊗$\odot$. For *J* ≈ 1 × 10^8^ A cm^−^^2^ (Extended Data Fig. 2a), the ⊗$\odot$ DWs are blocked in region A for the 3D twisted ribbon with *ζ* = +11° (region A, left half) and −11° (region B, right half). By contrast, $\odot$⊗ DWs pass through region A but are stuck in region B (*ζ* = −11°), as shown in Extended Data Fig. 2a. By contrast, for the twisted ribbon having *ζ* = −11° (region A, left half) and +11° (region B, right half) and vice versa (Extended Data Fig. 2c). An example for an intermediate value of *J* is shown in Extended Data Fig. 2b,d.

### Geometrical chiral-twist-dependent local exchange energy

To illustrate how the local exchange energy *E*_ex_ is affected by the chiral twist, schematic illustrations are shown in Fig. 4. The arrows on the 3D twisted ribbons represent the local magnetizations **M**. that is, red: $\odot$ (up domain) and blue: $\otimes$ (down domain) and green: DW. Here we consider the untilted DW case (*χ* = 0) only, for simplicity. Note that 3D twisted ribbons have two distinct faces, upper and lower, as the 3D twisted ribbons are mathematically orientable (but a Möbius strip is not orientable, that is, it has only one face). Figure 4a,b shows how the orthogonal unit vector set $\{\hat{{\bf{p}}},\hat{{\bf{r}}},\hat{{\bf{u}}}\}$ (thick black arrows) at the edge of the 3D twisted ribbon rotates around as the ribbon gets twisted by *ζ* = −180° (left-handed chirality) or *ζ* = +180° (right-handed chirality). The orientation of **M** can be defined with respect to either the upper or the lower face. Here the upper face at the left end of the ribbon (that is, *x* = 0) has been chosen to define the orientation of **M**. For example, the $\odot$⊗ DW configuration corresponds to *θ* = 0 ($\odot$) and π ($\otimes$) that are defined with respect to the normal vector $\hat{{\bf{u}}}$ on the upper face when the domains are at the left end.

As the ribbon gets twisted by π, the upper face faces up at the left end of the ribbon, rotates around and then faces down at the right end of the ribbon. Importantly, although **M** does not rotate at all, a DW can be created just by twisting the ribbon if the DW width (π*Δ*) equals the twisting length (π/*ξ*) by a π-rotation, as shown in Fig. 4a,b. In this case, **M** rotates in the opposite direction as the ribbon twists around its length direction $\hat{{\bf{p}}}$, such that **M** within the DW rotates less than the case without any twist, that is, the gradient of **M** becomes smaller. If the DW width were perfectly identical to the length of the π-twisted region, then $\hat{{\bf{m}}}$ would be perfectly uniform within the DW, thus effectively equivalent to a single magnetic domain in the cartesian coordinate system while there are two domains with a DW in the curvilinear coordinate system. Note that the exchange energy is determined by the magnetization gradient in the cartesian coordinate system. This happens when the DW is a Bloch-type wall with either (1) *ψ* = π/2 and the ribbon twists around in the right-handed way (*ζ* > 0) or (2) *ψ* = 3π/2 and the ribbon twists around in the left-handed way (*ζ* < 0). This describes the case that **M** remains the same when the arrow colour changes in Fig. 4a,b. In these cases, the local exchange energy per unit volume *ϵ*_ex_ is small and is the same as that for a single domain in a flat strip as the gradient of **M** is zero.

By contrast, if **M** rotates in the same direction as the ribbon is twisted around, **M** has to rotate around twice, that is, 2π to form a DW as the ribbon gets twisted by π. This happens when the DW is a Bloch-type wall either having (1) *ψ* = 3π/2 and the ribbon twists around in the right-handed way (*ζ* > 0), or (2) *ψ* = π/2 and the ribbon twists around in the left-handed way (*ζ* < 0). In these cases, *ϵ*_ex_ is large as the gradient of **M** is large. These show that *ϵ*_ex_ is determined by the geometrical chirality of the 3D twisted ribbon and the chirality of the Bloch-type walls. By contrast, for the untilted Néel-type walls (*ψ* = 0 or π), *ϵ*_ex_ in the DW is not affected by twisting the ribbon, thereby equaling that for a DW in a flat strip as **M** in DW rotates in the direction orthogonal to what the ribbon twisted around. In this case, *ϵ*_ex_ is intermediate between the two cases above.

Combining all the cases above, we can readily find that an effective DMI field **H**_tor_ is induced by the interplay between the chiral twist and the helical Bloch-type wall chirality. The orientation of **H**_tor_ is determined by the DW configuration, and the sign of *ζ* such that the torsional torque **τ**_tor_ = −*γ***M** × **H**_tor_ results in a geometrical chirality and DW-configuration-dependent DW motion by current as observed in our experiments. Note, for clarification, that the interface DMI stabilizes a chiral Néel-type DW structure but the geometrical twisting favours a chiral Bloch-type wall when the DW is untilted. Thus, the actual structure of the moving DW lies somewhere in between.

Note that the above cases describe only untilted DWs for which case a DW has a tilting angle *χ* = 0. When *χ* ≠ 0, the situation becomes more complicated, and the details are discussed in Supplementary Note 2. A comprehensive summary of all the cases is presented in Extended Data Table 1.

### Effective interface and twist-induced Dzyaloshinskii–Moriya interaction fields

The effective interface-induced DMI fields *H*_DM_≈1 kOe are extracted by fitting the *v*–*H*_*x*_ curves shown in Extended Data Fig. 8a from which we find *D* = *M*_s_*H*_DM_*Δ* ≈ 0.52 erg cm^−2^ (see Supplementary Note 5 for details). The twist-induced effective field *H*_tor_ = *A*_ex_/(*bM*_s_*Δ*) ≈ 126 Oe is obtained from *A*_ex_ = 34 pJ m^−1^, *M*_s_ = 520 emu cm^−3^, *Δ* = 4 nm, *L* = 1 μm, *ζ* = 11° and *ξ* = *ζ*/*L* = 0.19 μm^−1^. Note that *H*_tor_ plays a key part in the chiral-twist-dependent current-driven DW motion. From these, we find that the effective curvilinear chiral-geometry-induced DMI constant *D*_tor_ = 2*ξA*_ex_ = 6.5 × 10^−3^ erg cm^−2^. Here, *M*_s_ is experimentally measured, whereas the value of *A*_ex_ is estimated by interpolation from literature values^40^ for Co (56 pJ m^−1^) and Ni (15 pJ m^−1^) and the film stacks (3 Å Co/7 Å Ni/3 Å Co). By contrast, the value of *ζ* that is used is the nominally designed value, whereas the values of *Δ* and *L* are obtained by fitting *v*–*J* curves in Fig. 3. The fitted *Δ* = 4 nm is different from what is estimated (10 nm) from *A*_ex_ and *K*_eff_ above. Note that there are significant uncertainties in the determination of *Δ*. *Δ* =4 nm agrees with those from similar published film stacks that show *Δ* = 4.3 nm for 30 Pt/3 Co/7 Ni/1.5 Co (ref. ^41^). The fitted *L* = 1 μm is lower than the designed value *L* ≈ 5 μm (Supplementary Fig. 14). This difference may be because of the following. First, the effective *L* may be smaller than the designed *L* because the gradient of angle variation along the 3D twisted ribbon can deviate from linearity, as shown in Supplementary Fig. 14. Second, the fabricated structures are slightly off from the designed ideal 3D twisted ribbons apart from the nonlinear twisting angle variation. Third, our analytical model has been developed using approximations based on *ξw* ≫ 1 (where *w* is the width of the 3D twisted ribbon) for simplicity to capture the underlying physics, as discussed in detail in Supplementary Note 2. Fourth, hidden imperfections or pinning sites may amplify the chiral-twist-dependent current-driven DW motion. Larger geometrical chirality-induced DW motion in experiment than that in model predictions have also been observed in curved wires^19^ and Y-shaped wires^20^ on flat surfaces. Most importantly, note that all trends of chiral-twist-dependent CIDWM agree well with the model predictions although there are quantitative differences between the experiment and the analytical model.

### Torsional fields and torsional torques

We developed an analytical model to understand our observations. A comprehensive discussion is included in Supplementary Note 2.

### Current-driven chiral DW motion with reversed DW chirality and/or opposite SHE-induced SOT in twisted ribbons

Our model shows that the twisting effect on the current-induced DW motion is determined not only by the geometrical chiral twist but also by the chirality of a Néel-type DW. To verify this prediction experimentally, we prepared twisted ribbons that are coated with two distinct film stacks that have reversed DW chirality and/or opposite SHE-induced SOT by placing a thick Pt layer on top of Pt/Co/Ni/Co and tuning the respective thicknesses of the Co and Pt layers^18^. We find that the measured current-induced DW motions in devices with reversed DW chirality and/or opposite SOT are significantly slowed down because of competition between the bottom and top interface DMIs and SOTs apart from the velocity reduction in 3D twisted ribbons compared with flat strips. This makes these experiments highly challenging for reliable measurements of DW motions that are much more susceptible to DW pinning–depinning at the greater number of imperfections in the 3D twisted ribbons. We find that the twisting effect can be masked by this DW pinning–depinning, as discussed below.

Note that the chirality of a Néel-type DW is determined by the competition between the DMIs induced from the bottom and top interfaces. When the DMI at the bottom Pt/Co interface is larger than that at the top Co/Pt interface, the DW is left-handed and when it is smaller, the DW is right-handed. The DMI strength is typically proportional to the Co layer thickness. By contrast, the SHE-induced SOT is positive when the SHE from the bottom Pt layer is larger and negative when the SHE from the bottom Pt layer is smaller than that from the top Pt layer. The SHE is proportional to the Pt layer thickness when the Pt layer is not much thicker than the spin diffusion length of Pt. Note that the film stack used in Figs. 2–4 has a left-handed DW chirality and a positive SHE-induced SOT.

**Extended Data Fig. 4:**
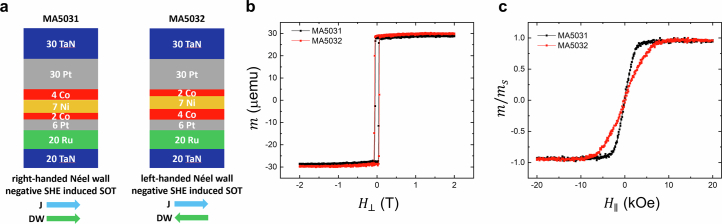
Magnetic thin films having reversed DW and spin current chiralities. **a**, Film stack for newly optimized films used to test the impact of the Néel DW chirality inversion. Thicknesses in Å. Sketches showing the Pt interface layers (top/bottom, relative to the Co/Ni/Co ferromagnetic layer) from where the SHE and DMI originate. The arrows display the direction of DW motion under a left-to-right current direction. **b**,**c**, Hysteresis loops of the FM film stack deposited on the substrate, measured by VSM along the easy axis (out-of-plane, **b**) and the hard axis (in-plane, **c**).

First, the following film stacks were grown on flat Si/SiO_*x*_ substrates to check the DW chirality and the SHE-induced SOT (Extended Data Fig. 4):

- 6 Pt/2 Co/7 Ni/4 Co/30 Pt (sample ID MA5032): left-handed DW chirality and negative SHE-induced SOT and
- 6 Pt/4 Co/7 Ni/2 Co/30 Pt (sample ID MA5031): right-handed DW chirality and negative SHE-induced SOT.

Although the PMA is decent in these films, the anisotropy strength is smaller than for the 30 Pt/3 Co/7 Ni/3 Co films, thereby limiting the application of high current densities due to DW nucleation. The *v*–*H*_*x*_ curves are useful to determine the chirality of the interface DMI-induced chirality in Néel-type DWs and the sign of the SHE-induced SOT. The fields *H*_CR_ in the *v*–*H*_*x*_ curves, in which the velocity crosses through zero along the *H*_*x*_ axis are positive and negative for $\odot$⊗ and ⊗$\odot$ DWs, respectively, the DW chirality is left-handed, and in which the velocity crosses through zero along the *H*_*x*_ axis are negative and positive for $\odot$⊗ and ⊗$\odot$ DWs, respectively, the DW chirality is right-handed. By contrast, when the signs of the slope are negative and positive for $\odot$⊗ and ⊗$\odot$ for positive currents *J* > 0, respectively, the sign of SHE-induced SOT is positive, and when the signs of the slope are positive and negative for $\odot$⊗ and ⊗$\odot$ for positive currents *J* > 0, respectively, the sign of SHE-induced SOT is negative. For *J* < 0, the slope signs are reversed because of the reversed spin polarization in spin current, whereas *H*_CR_ does not change.

**Extended Data Fig. 5:**
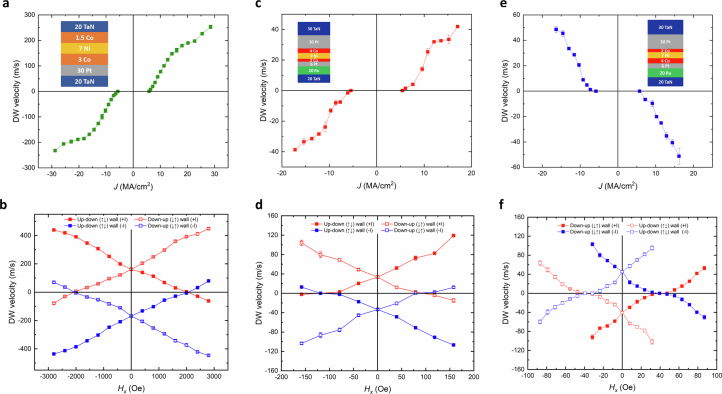
Current induced motion of DWs with different DW chiralities and SHE induced SOT from flat strips on Si/SiO_2_ substrates. **a**,**c**,**e**, $v-J$ curves, **b**,**d**,**f**, $v-{H}_{x}$ curves from 30 Pt/3 Co/7 Ni/1.5 Co (**a**,**b**), 6 Pt/2 Co/7 Ni/4 Co/30 Pt (**c**,**d**), and 6 Pt/4 Co/7 Ni/2 Co/30 Pt (**e**,**f**). The corresponding chiralities for Néel-type DWs and the SHE induced SOT are left-handed/positive SOT (**a**,**b**), right-handed/negative SOT (**c**,**d**), and left-handed/negative SOT (**e**,**f**). Error bars represent standard deviation.

For 30 Pt/3 Co/7 Ni/3 or 1.5 Co that has been used for the twisting-dependent experiments in our paper, the DW is a left-handed Néel-DW and the SOT is positive, as shown in Extended Data Fig. 5a,b, thereby showing that the DW moves along the current flow direction in the absence of *H*_*x*_ (Extended Data Fig. 5a). Note that, for a given *J*, the DW velocities in the flat strips in Extended Data Fig. 5 are significantly larger than those from a ribbon having *ζ* = 0° shown in Fig. 3a. This shows that some imperfections and pinning sites in the ribbons significantly increase the threshold current density *J*_c_, thereby reducing the DW velocity overall.

Right-handed Néel-type DWs (reversed DW chirality) are found in flat strips formed from the film stack 6 Pt/2 Co/7 Ni/4 Co/30 Pt on Si/SiO_*x*_, as shown in Extended Data Fig. 5d (*H*_CR_ < 0 for $\odot$⊗ and *H*_CR_ > 0 for ⊗$\odot$). As the top Pt layer (30 Å) is thicker than the bottom Pt layer (6 Å), the sign of the SHE-induced SOT is reversed and negative, thereby leading to positive and negative slopes for for *J* > 0 and negative and positive slopes for DWs, respectively, for *J* < 0, as shown in Extended Data Fig. 5d. Consequently, the DW moves along the current flow direction in the absence of *H*_*x*_ (Extended Data Fig. 5c). Note that the DW velocities are low because of the competition between the interface-induced DMIs from the top and bottom interfaces, as compared with those with left-handed chirality and positive SHE-induced SOT from a single Pt layer.

To investigate how the SOT interacts with the geometrical twisting, we have additionally prepared twisted ribbons coated with the film stack 6 Pt/4 Co/7 Ni/2 Co/30 Pt that has a left-handed Néel-type DW and a negative SHE-induced SOT. As shown in Extended Data Fig. 5f, *H*_CR_ > 0 for $\odot$⊗ and *H*_CR_ < 0 for ⊗$\odot$, whereas positive and negative slopes for $\odot$⊗ and, respectively, for *J* > 0, and negative and positive slopes for $\odot$⊗ and ⊗$\odot$, respectively, for *J* < 0. Consequently, the DWs move along the electron flow direction in the absence of *H*_*x*_ as shown in Extended Data Fig. 5e. As for the right-handed Néel-type DWs and negative SHE-induced SOT, the overall DW velocities are significantly reduced.

**Extended Data Fig. 6:**
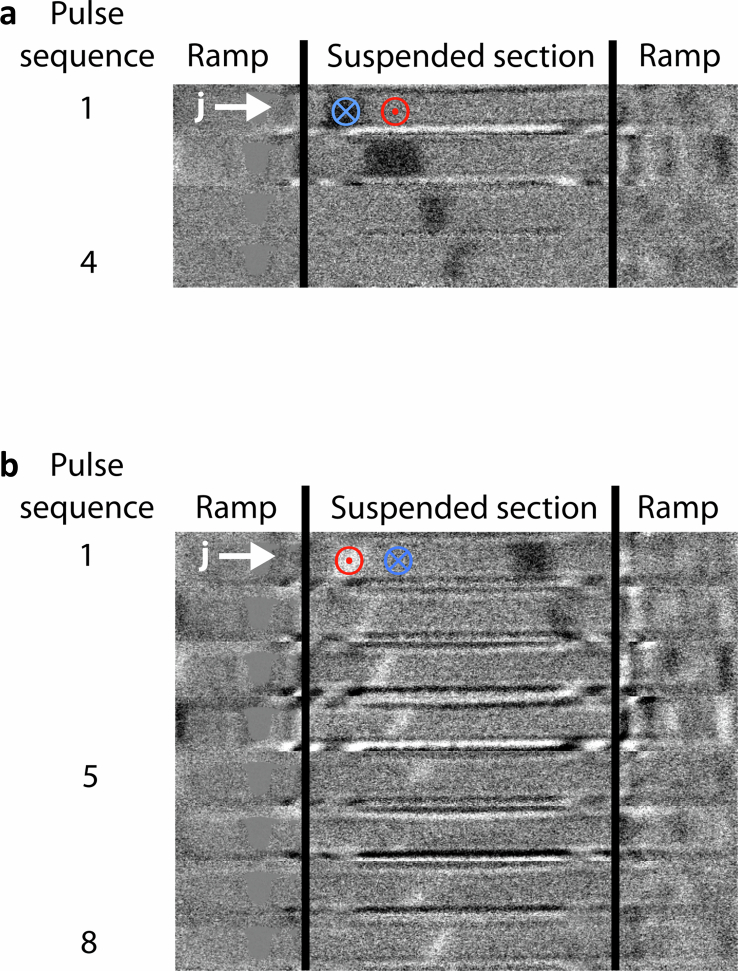
Displacement of right-handed DWs by negative SHE induced SOT. The SOT is induced from the top Pt layer in the film stack 6 Pt/2 Co/7 Ni/4 Co/30 Pt as a function of the number of pulse sequences (vertical axis) for a ⊗$\odot$ (**a**) and $\odot$⊗ (**b**) along the left-handed chiral twist region (*ζ* = −11°) in a twisted ribbon. Cumulative pulse duration is 3 and 6 μs, respectively.

**Extended Data Fig. 7:**
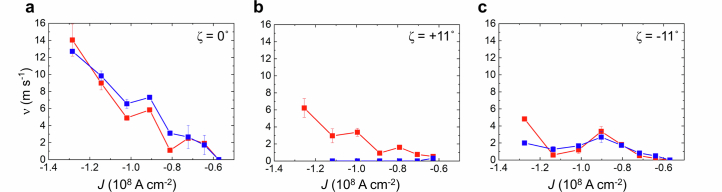
DW velocity versus current density *J* (<0) measured from twisted ribbons with left-handed DW chirality and negative SHE induced SOT. The ribbons have **a**, *ζ* = 0 (untwisted), **b**, +11° (right-handed) and **c**, −11° (left-handed), respectively, that are coated with 6 Pt/4 Co/7 Ni/2 Co/30 Pt. This film stack has a left-handed DW chirality from the bottom Pt/Co interface and negative SHE induced SOT from the top Pt layer. Red and blue solid lines and symbols correspond to $\odot$⊗ and ⊗$\odot$ DWs, respectively. Error bars represent the average deviation from the mean.

**Extended Data Fig. 8:**
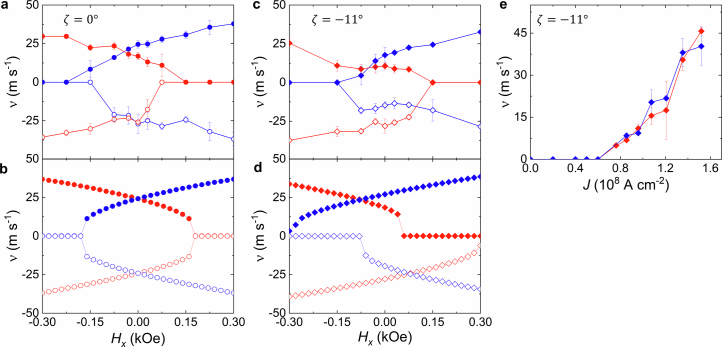
CIDWM in FM 3D twisted ribbon under in-plane longitudinal fields *H*_*x*_ and SAF 3D twisted ribbon. Red (blue) represent $\odot$⊗ (⊗$\odot$) DW configurations, respectively, and circle and diamond symbols represent *ζ* = 0° and −11°, respectively. **a,c** Experimentally measured DW velocity as a function of the applied in-plane field ${H}_{x}$ for both DW configurations in FM 3D twisted ribbons when current flows along +$x$ (solid symbols) and –*x* (open symbols) direction over sections with *ζ* = 0° (**a**) and −11° (**c**). **b**,**d**, Analytical model calculations to simulate the experimental data in **a**,**c**, respectively. The parameters for the calculations are ${t}_{p}=50$ ns, $\varDelta =2$ nm, $\alpha =0.1$, ${H}_{k}=2$ kOe, ${H}_{{DM}}=-\,1$ kOe for $\odot$⊗ and 1 kOe for ⊗$\odot$, $J=3.15\times {10}^{7}$ A cm^−2^ (${H}_{{SH}}=0.2$ kOe) and $J=-\,3.15\times {10}^{7}$ A cm−2 (${H}_{{SH}}=-\,0.2$ kOe), ${M}_{s}=520$ emu cm^−3^, $L=1$ μm ($\xi =0.19$ μm^−1^), ${A}_{{ex}}=3.4\times {10}^{-6}$ erg cm^−1^, $V=2.1\times {10}^{5}$ erg cm^−3^, ${q}_{0}=2$ nm (**d**), and ${q}_{{\rm{shift}}}=0$ nm. **e**, Experimental ${\rm{\nu }}-J$ plot for *ζ* = −11° in a SAF 3D twisted ribbon. Error bars represent the average deviation from the mean.

First, a 6 Pt/2 Co/7 Ni/4 Co/30 Pt film that has a reversed DW chirality (right-handed) was grown on a left-handed twisted ribbon with *ζ* = −11° that corresponds to the left half of the twisted ribbon. As shown in Extended Data Fig. 6, the DW velocity for (2.49 ± 0.53 m s^−1^) is larger than that for $\odot$⊗ (1.29 ± 0.20 m s^−1^) at *J* = 6.4 × 10^7^ A cm^−2^. This agrees with the prediction of our analytical model that the torsional torque is determined not only by the geometrical chiral twist but also by the DW chirality, as shown in Extended Data Table 2. Next, current-induced DW motions are measured from non-twisted and right-handed and left-handed twisted ribbons that were coated with 6 Pt/4 Co/7 Ni/2 Co/30 Pt films. Note that this film stack has a left-handed DW chirality and a negative SHE-induced SOT such that the DW moves along the electron flow direction (opposite to the current flow direction). Thus, *J* < 0 that is used during the measurements leads to *v* > 0. Note that DW velocities are measured only in the left-half region of the twisted ribbon. DW velocities for $\odot$⊗ are nearly identical to those for ⊗$\odot$ for an untwisted ribbon as anticipated from our model (Extended Data Fig. 7). By contrast, a $\odot$⊗ DW moves faster than a ⊗$\odot$ DW for the right-handed chiral twist (Extended Data Fig. 7b). This agrees with the model prediction (Extended Data Table 3). For a left-handed chiral twist, the DW velocity differences between the two DW configurations are not large (Extended Data Fig. 7c). This may be because DW velocities are overall very small (<5 m s^−1^), thereby being more sensitive to pinning–depinning because of imperfections that can mask the torsional torque effect in the devices with reversed DW chiralities and SHE-induced SOT. Further improved fabrication processes that minimize such DW pinning–depinning will allow for clearer differences.

## Online content

Any methods, additional references, Nature Portfolio reporting summaries, source data, extended data, supplementary information, acknowledgements, peer review information; details of author contributions and competing interests; and statements of data and code availability are available at 10.1038/s41586-024-08582-8.

## Supplementary information

Supplementary Information This file contains Supplementary Notes 1–6 and Supplementary References.

## Figures and Tables

**Extended Data Table 1:**
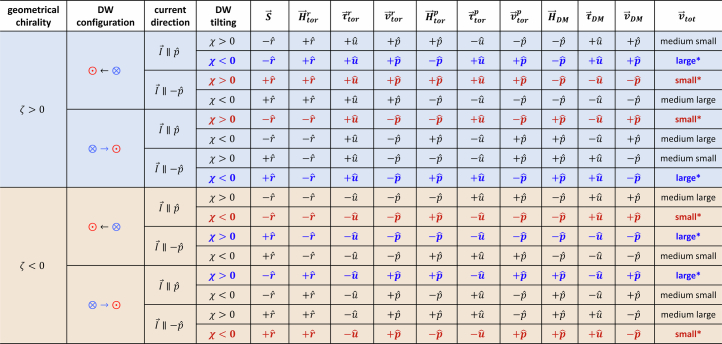
Summary of orientations of spins $\overrightarrow{{\bf{S}}}$ in spin-orbit interaction induced spin current, effective fields $\overrightarrow{{\bf{H}}}$, effective torques $\overrightarrow{{\boldsymbol{\tau }}}$ and DW velocities $\overrightarrow{{\bf{v}}}$ for various chiral twists, DW configurations and chiralities, current direction $\overrightarrow{{\bf{I}}}$, and DW tilting angle ${\boldsymbol{\chi }}$ for twisted ribbons coated with film stack having a “left-handed DW chirality” and a “positive SHE induced SOT”

The orientation of $\overrightarrow{{\bf{S}}}$ is determined by a positive spin Hall angle from the Pt layer. The DW velocity ${\overrightarrow{{\bf{v}}}}_{tot}={\overrightarrow{{\bf{v}}}}_{tor}^{r}+{\overrightarrow{{\bf{v}}}}_{tor}^{p}+{\overrightarrow{{\bf{v}}}}_{DM}$ can be divided into three components, ${\overrightarrow{{\bf{v}}}}_{tor}^{r},\,{\overrightarrow{{\bf{v}}}}_{tor}^{p},$ and ${\overrightarrow{{\bf{v}}}}_{DM}$ that denote the DW velocities induced by ${\overrightarrow{{\bf{H}}}}_{tor}^{r}$ (the parallel component of ${\overrightarrow{{\bf{H}}}}_{tor}$ with $\hat{{\bf{r}}}$), ${\overrightarrow{{\bf{H}}}}_{tor}^{p}$ (the parallel component of ${\overrightarrow{{\bf{H}}}}_{tor}$ with $\hat{{\bf{p}}}$), and ${\overrightarrow{{\bf{H}}}}_{DM}$ (interfacial DMI field), respectively. Rows in bold blue and red correspond to large and small ${\overrightarrow{{\bf{v}}}}_{tot}$, respectively. The corresponding experimental results shown in Figs. 3 and 5 are highlighted by an asterisk (*) that show enhanced and reduced DW velocity by the chiral twist. The corresponding film stack with left-handed DW chirality and positive SHE induced SOT is 30 Pt/3 Co/7 Ni/3 Co. See Supplementary Note 2 for more details.

**Extended Data Table 2:**
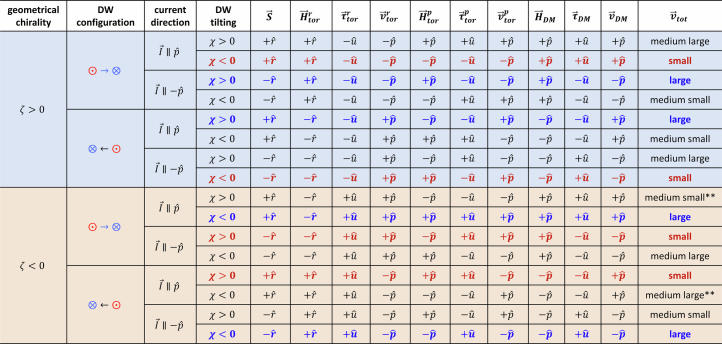
Summary of orientations of $\overrightarrow{{\bf{S}}}$, $\overrightarrow{{\bf{H}}}$, $\overrightarrow{{\boldsymbol{\tau }}}$ and $\overrightarrow{{\bf{v}}}$ for “right-handed Néel-type DWs” and “negative SHE induced SOT” with different chiral twists, DW configurations, $\overrightarrow{{\bf{I}}}$, and ${\boldsymbol{\chi }}$

The orientation of $\overrightarrow{{\bf{S}}}$ is determined by a negative SHE induced SOT from the top Pt layer. Right-handed Néel-type DWs are induced by the top Co/Pt interface. Rows in bold blue and red correspond to large and small ${\overrightarrow{{\bf{v}}}}_{tot}$, respectively. The corresponding experimental results shown in Extended Data Fig. 6 are highlighted by double asterisks (**) that show enhanced and reduced DW velocity by the chiral twist. The corresponding film stack with left-handed DW chirality and positive SHE induced SOT is 6 Pt/2 Co/7 Ni/4 Co/30 Pt. See the detailed discussion in the Methods.

**Extended Data Table 3:**
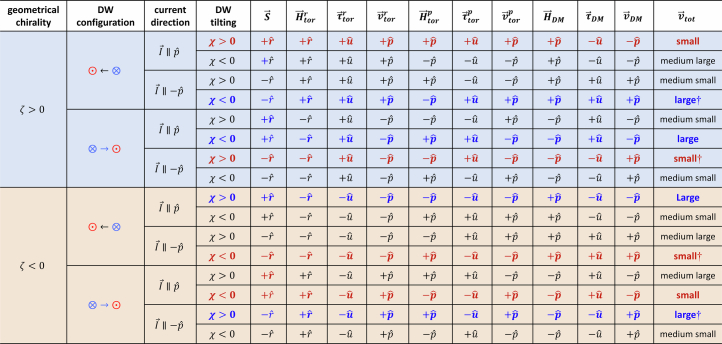
Summary of orientations of $\overrightarrow{{\bf{S}}}$, $\overrightarrow{{\bf{H}}}$, $\overrightarrow{{\boldsymbol{\tau }}}$ and $\overrightarrow{{\bf{v}}}$ for “left-handed Néel-type DWs” and “negative SHE induced SOT” with different chiral twists, DW configurations, $\overrightarrow{{\bf{I}}}$, and ${\boldsymbol{\chi }}$

The orientation of $\overrightarrow{{\bf{S}}}$ is determined by a negative SHE induced SOT from the top Pt layer. Right-handed Néel-type DWs are induced by the top Co/Pt interface. Rows in bold blue and red correspond to large and small ${\overrightarrow{{\bf{v}}}}_{tot}$, respectively. The corresponding experimental results shown in Extended Data Fig. 7 are highlighted by daggers (†) that show enhanced and reduced DW velocity by chiral twist. The corresponding film stack with left-handed DW chirality and positive SHE induced SOT is 6 Pt/4 Co/7 Ni/2 Co/30 Pt. See the detailed discussion in the Methods.

## Data Availability

Source data are available from the corresponding authors on request.

## Code availability

The code utilized for data processing can be made available from the corresponding authors upon request.
