# Supplementary material for: Interplay of geometrical and spin chiralities in 3D twisted magnetic ribbons
Source: Nature. 2025 Feb 26;639(8053):67–72. doi: 10.1038/s41586-024-08582-8 (PMC11882454; doi:10.1038/s41586-024-08582-8)
Supplement: Supplementary file 1 — This file contains Supplementary Notes 1–6 and Supplementary References. [file 41586_2024_8582_MOESM1_ESM.docx]

**Supplementary Information for**

**Interplay of geometrical and spin chiralities in twisted magnetic ribbons**

André Farinha^1,2^, See-Hun Yang^1†^, Jiho Yoon^1^, Banabir Pal^1^, and Stuart S. P. Parkin^1,2†^

^1^Max Planck Institute of Microstructure Physics, 06120, Halle, Germany

^2^Institute of Physics, Martin Luther University, Halle-Wittenberg, 06120, Halle, Germany

^†^Email: [sehuyang@mpi-halle.mpg.de](mailto:sehuyang@mpi-halle.mpg.de), [stuart.parkin@mpi-halle.mpg.de](mailto:stuart.parkin@mpi-halle.mpg.de)

**List of Contents**

**Supplementary Note 1: Fabrication of 3D twisted magnetic ribbons**

**Supplementary Note 2: Analytical model for current-driven chiral domain wall motion in 3D** **twisted magnetic ribbons**

**Supplementary Note 3: Magnetostatic interaction in chiral domain walls on 3D twisted ribbons**

**Supplementary Note 4: Additional device characterization**

**Supplementary Note 5: Hysteresis loops and micromagnetic parameters**

**Supplementary Note 6: Twisted angle longitudinal profiles**

**Supplementary References**

**Supplementary Note 1: Fabrication of 3D twisted magnetic ribbons**

Our device design simplifies the fabrication process that comprises just two main steps: the fabrication of the 3D polymeric structures and the film deposition. Light from a fs-laser is passed through a transparent substrate and is thereby tightly focused onto a negative-tone photoresist under the needed exposure conditions to trigger two-photon absorption, leading to a 3D-localized radical polymerization chain-reaction. This allows freeform 3D fabrication by scanning the laser beam focus within the photoresist volume. The fabrication is performed layer by layer. Within a layer each scan is carried out as closely as reliably possible to the next one with a spacing of 25 nm in X and Z that corresponds to at least 1/4 of the minimum feature (voxel) size. This allows us to achieve very smooth surfaces. SEM images of the fabricated structures show that the surfaces are smooth in general except the twisting and ramp sections that have some steps. Such steps serve as pinning centers for CIDWM. The multi-photon lithography (MPL) fabrication yield is better than 92$\%$ from ~70 devices.


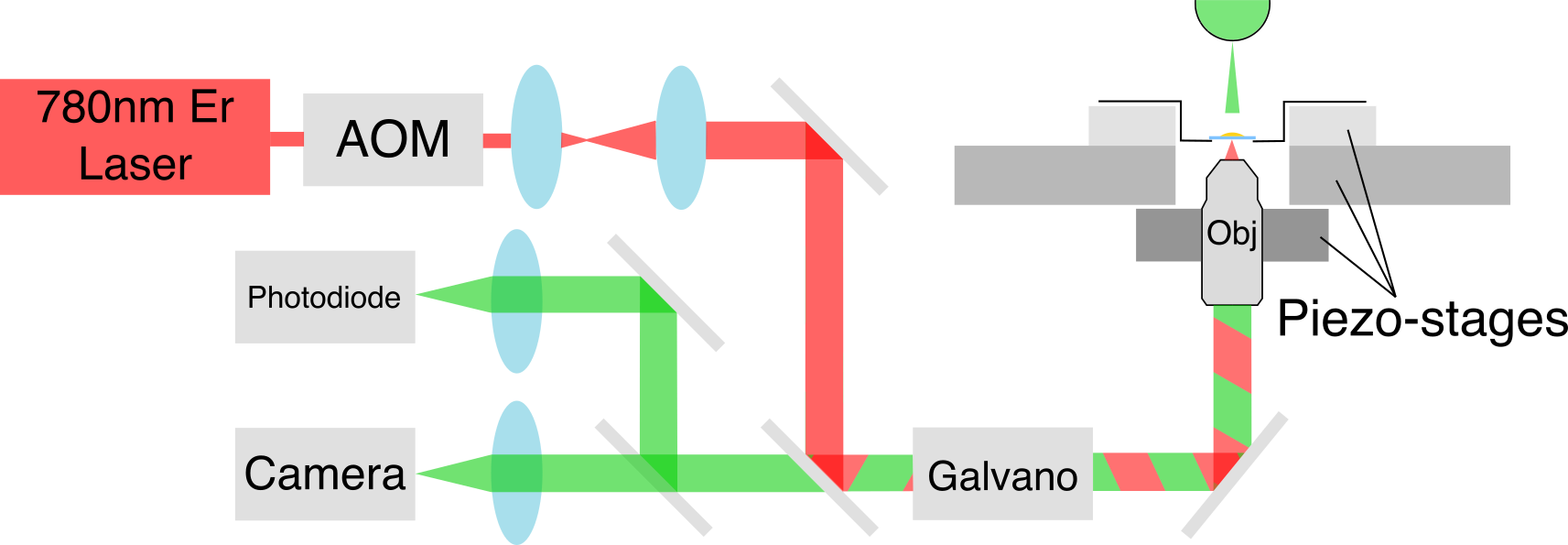


**Figure S1 | Schematic diagram of multiphoton lithography setup.**


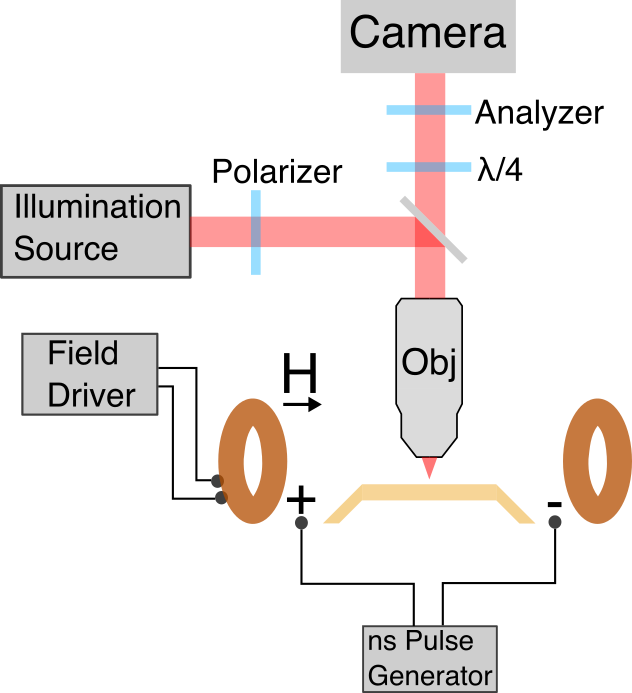


**Figure S2 | Schematic diagram of Kerr microscope setup**

**Supplementary Note 2: Analytical model for current-driven chiral domain wall motion in 3D** **twisted magnetic ribbons**

To understand the current-driven motion of chiral domain walls (DWs) in 3D twisted magnetic ribbons, we developed an analytical model to capture the underlying interplay between chiralities that determine the dynamic of chiral DWs. We find that micromagnetic simulations are challenging to be applied to our systems due to non-straightforward meshing and large dimensions of 3D twisted ribbons. The developed analytical model is an extended $q-\phi$ model that further considers the contributions from DW tilting, geometrical chirality, and magnetostatic interactions. Note that there are limitations due to the approximations and assumptions such as uniform $\phi$ all over the magnetic structures.


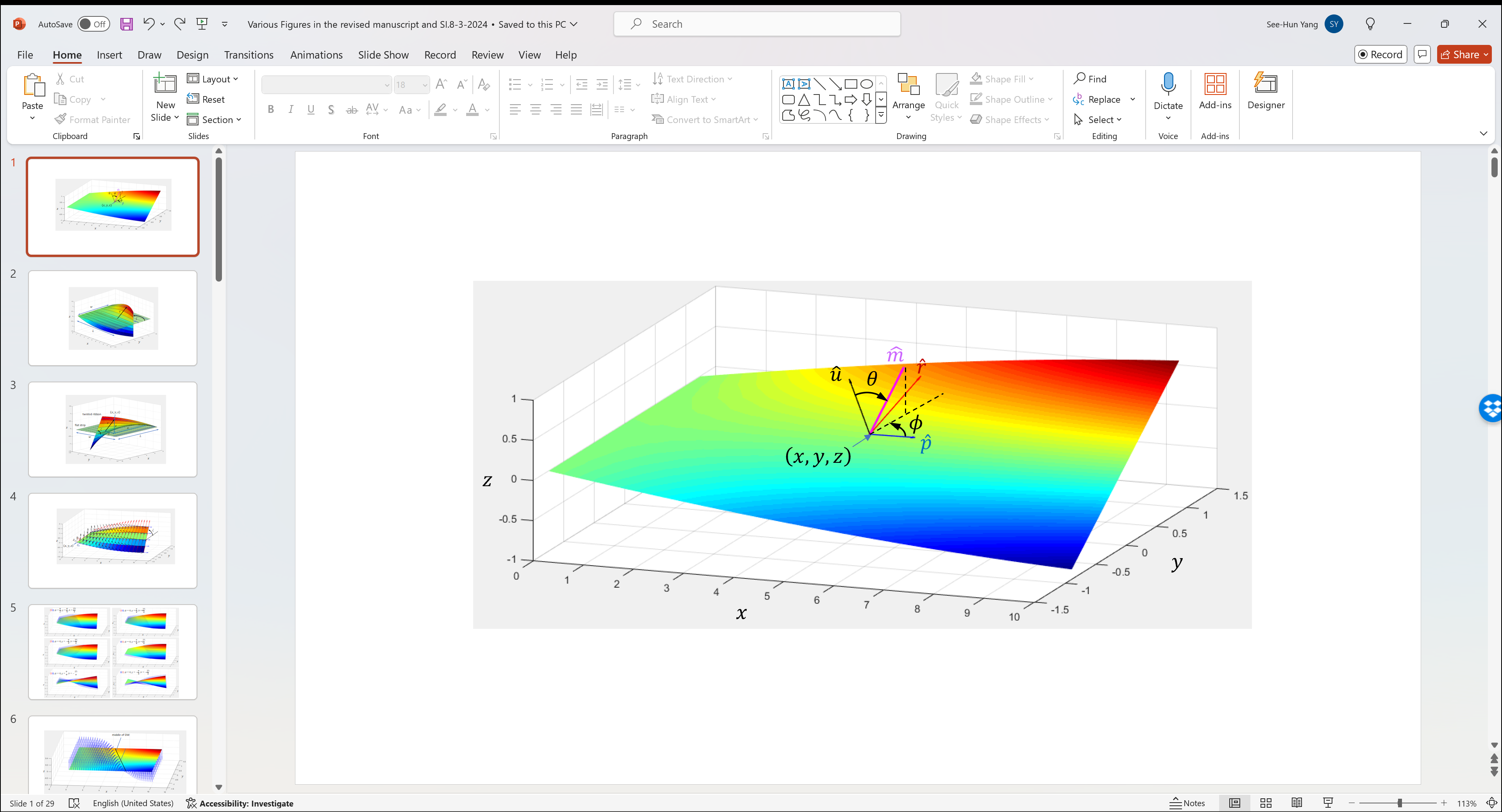


**a**


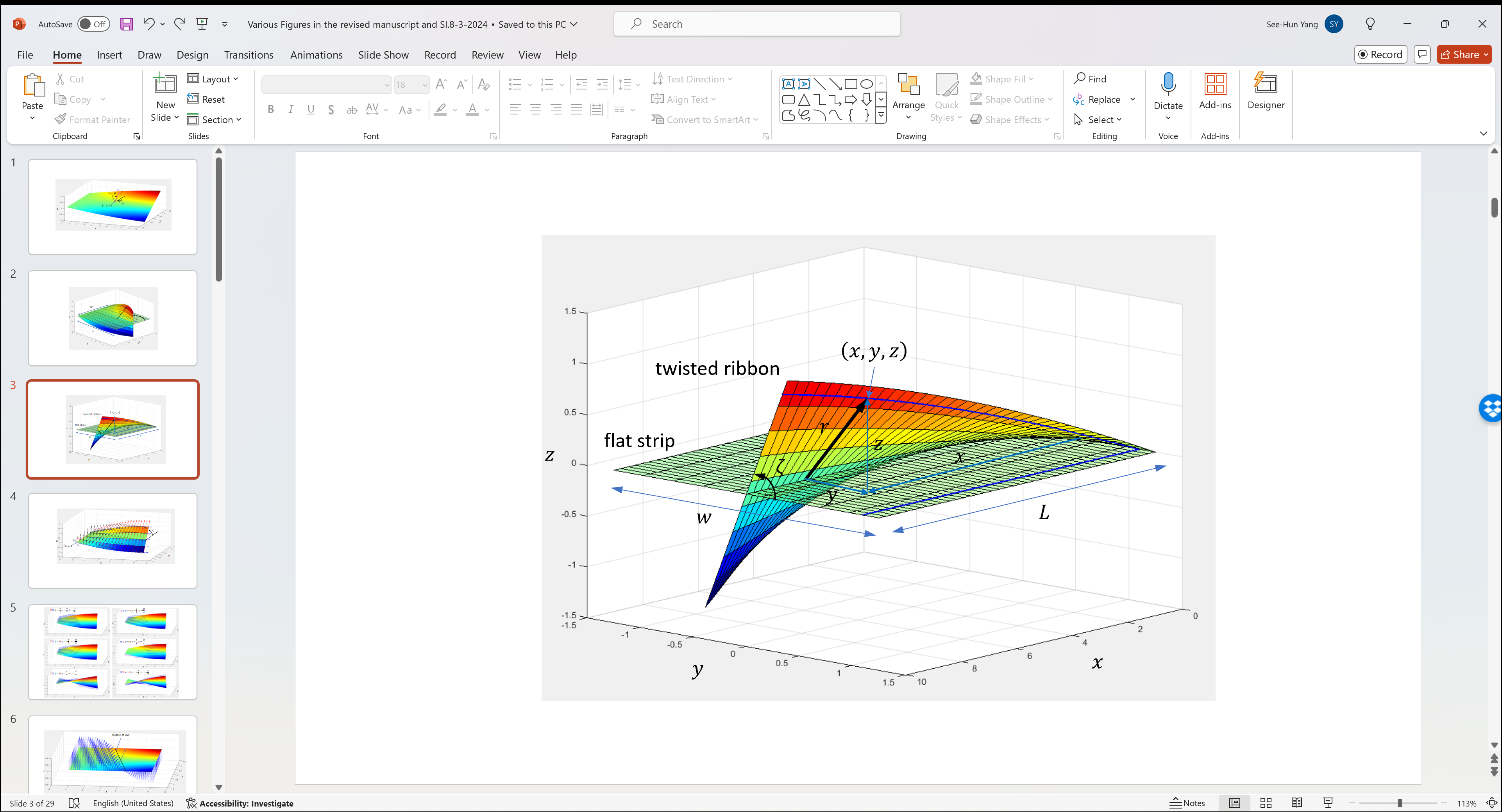


**b**


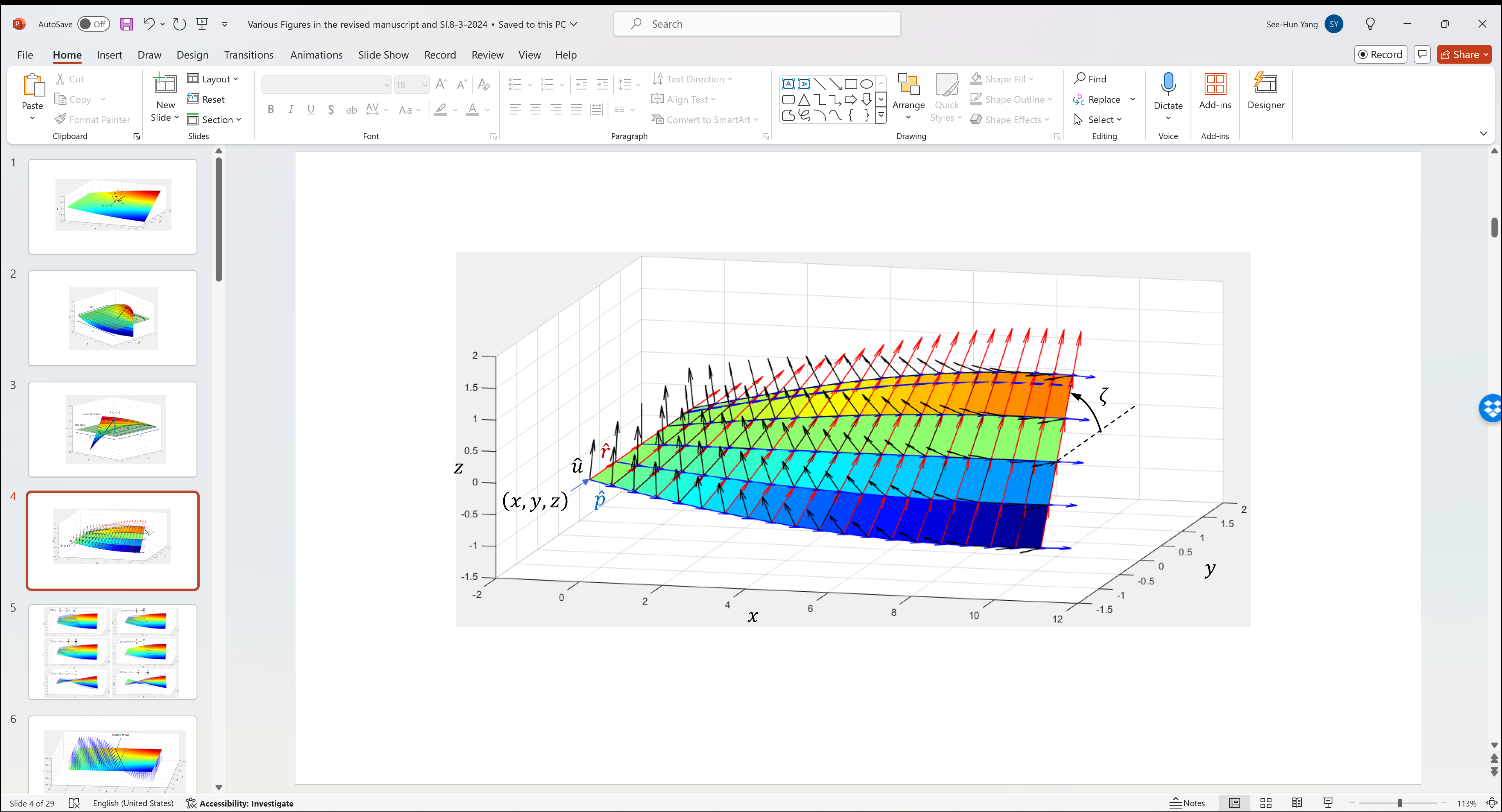


**c**

**Figure S3** | **Calculated illustration of cartesian coordinates, curvilinear coordinates, and orthonormal unit vector set** **a**, Magnetization $\hat{m}$ at $\left( x,y,z \right)$ defined with respect to the orthogonal unit vector set $\left\{ \hat{p},\hat{r},\hat{u} \right\}$ with a polar angle $\theta$ and a azimuthal angle $\phi$. **b**, A point at $\left( x,y,z \right)$ on a 3D twisted ribbon having width $w$, length $L$ and twisting angle $\zeta$. The helicoid wire is distanced from the middle straight line by $r=\sqrt{y^{2}+z^{2}}$. **c**, Orthogonal unit vector set $\left\{ \hat{p},\hat{r},\hat{u} \right\}$ at a point $\left( x,y,z \right)$ on 3D twisted ribbon with width $w=2.5$ twisting angle $\zeta$ over $L=10$.

Firstly, a 3D twisted magnetic ribbon can be described by parametric equations. When the twisting axis, around which the magnetic wire is twisted, is along $x$: $x=p, y=r\cos\frac{p}{b}$ and $z=r\sin\frac{p}{b}$, where $\left( x,y,z \right)$ are on a 3D twisted ribbon surface (see Fig. S3a). These constitute ribbon equation $\vec{\varsigma}=p\hat{x}+r\cos\frac{p}{b}\hat{y}+r\sin\frac{p}{b}\hat{z}$. The ranges of $p$ and $r$ are $0\leq p\leq L$ and $-\frac{w}{2}\leq r\leq\frac{w}{2},$ where $L$ and $w$ correspond to length and width of the ribbon, respectively. $b$ corresponds to the length over which the ribbon is twisted around per unit angle in radians. Hence, when a ribbon is twisted around by twisting angle $\zeta$ in radians over the twisted ribbon length $L$, i.e., $b=\frac{L}{\zeta}$. $b$ measures how much the ribbon is twisted such that the twist parameter $\xi=\frac{\vec{\varsigma}^{'}\times\vec{\varsigma}^{''}\cdot\vec{\varsigma}^{'''}}{\left| \vec{\varsigma}^{'}\times\vec{\varsigma}^{''} \right|^{2}}=\frac{x^{'''}\left( y^{'}z^{''}-y^{''}z^{'} \right)+y^{'''}\left( x^{''}z^{'}-x^{'}z^{''} \right)+z^{'''}\left( x^{'}y^{''}-x^{''}y^{'} \right)}{\left( y^{'}z^{''}-y^{''}z^{'} \right)^{2}+\left( x^{''}z^{'}-x^{'}z^{''} \right)^{2}+\left( x^{'}y^{''}-x^{''}y^{'} \right)^{2}}=\frac{-y^{'''}z^{''}+z^{'''}y^{''}}{\left( y^{'}z^{''}-y^{''}z^{'} \right)^{2}+{z^{''}}^{2}+{y^{''}}^{2}}=\frac{b}{b^{2}+r^{2}}$, which is introduced by analogy to the torsion parameter of space curves. Here $X^{'}=\frac{\partial X}{\partial p}$, $X^{''}=\frac{\partial^{2}X}{\partial p^{2}}$, and $X^{'''}=\frac{\partial^{3}X}{\partial p^{3}}$ ($X=x,y, z$. See Fig. S3b). A twisting angle $\zeta=\frac{L}{b}$ in which a ribbon is twisted over the length $L$ by the angle $\zeta$ (in radian) with a given $b$. Since the twist parameter $\xi=\frac{b}{b^{2}+r^{2}}=\frac{L\zeta}{r^{2}\zeta^{2}+L^{2}}$ ($r$ is the curvilinear coordinate perpendicular to ribbon length direction on the twisted ribbon. See Fig. S3b), using $L\sim4$ μm and $-1.25\leq r\leq1.25$ μm in our devices, $\xi$ is nearly linear with respect to $\zeta$ over $-11^{\circ}\leq\zeta\leq11^{\circ}$. This is because $b\gg r$ in our devices so that $\xi\approx\frac{1}{b}\equiv\frac{\zeta}{L}.$ These show that not only $\xi$ represents $\zeta$ well but also $\xi$ is nearly constant over the entire ribbon. Consequently, our twisted ribbons can be well-characterized by $\xi$ such that $b$ can be replaced by $\frac{1}{\xi}$, i.e. $\vec{\varsigma}=p\hat{x}+r\cos\xi p\hat{y}+r\sin\xi p\hat{z}$. The sign of $\xi$ corresponds to the chirality of ribbon: $\xi>0$ (right-handed) and $\xi<0$ (left-handed).

From the parametrized ribbon equation, orthogonal metric vectors can be derived to be $\vec{g}_{p}=\frac{\partial\vec{\varsigma}}{\partial p}=\left( 1,-r\xi\sin\xi p,r\xi\cos\xi p \right)$ and $\vec{g}_{r}=\frac{\partial\vec{\varsigma}}{\partial r}=\left( 0,\cos\xi p,\sin\xi p \right)$ at point $\left( x,y,z \right)$ on a twisted ribbon^1,2^. Note that $\vec{g}_{p}$ and $\vec{g}_{r}$ are tangential vectors, respectively, from which unit orthogonal tangential vectors $\hat{p}=\frac{\vec{g}_{p}}{\left| \vec{g}_{p} \right|}=\frac{1}{\sqrt{1+\xi^{2}r^{2}}}\left( 1,-r\xi\sin\xi p,r\xi\cos\xi p \right)$ and $\hat{r}=\frac{\vec{g}_{r}}{\left| \vec{g}_{r} \right|}=\left( 0,\cos\xi p,\sin\xi p \right)$ can be obtained. Then a unit normal vector $\hat{u}=\hat{p}\times\hat{r}=\frac{1}{\sqrt{1+\xi^{2}r^{2}}}\left( -\xi r,-\sin\xi p,\cos\xi p \right)$ can be derived so that we have an orthonormal vector set $\left\{ \hat{p},\hat{r},\hat{u} \right\}$ at $\left( x,y,z \right)$ on the twisted ribbon (see the Fig. S3c). With $\left\{ \hat{p},\hat{r},\hat{u} \right\}$, a unit magnetization $\hat{m}$ on twisted ribbon can be expressed by azimuthal angle $\phi$ and polar angle $\theta$ with respect to $\left\{ \hat{p},\hat{r},\hat{u} \right\}$ coordinate as follows (see Fig. S3a):

$$\hat{m}=\sin\theta\cos\phi\hat{p}+\sin\theta\sin\phi\hat{r}+\cos\theta\hat{u}$$

$$=\frac{\sin\theta\cos\phi-\xi r\cos\theta}{\sqrt{1+\xi^{2}r^{2}}}\hat{x}+\left( \sin\theta\sin\phi\cos\xi p-\sin\xi p\frac{r\xi\sin\theta\cos\phi+\cos\theta}{\sqrt{1+\xi^{2}r^{2}}} \right)\hat{y}+\left( \sin\theta\sin\phi\sin\xi p+\cos\xi p\frac{r\xi\sin\theta\cos\phi+\cos\theta}{\sqrt{1+\xi^{2}r^{2}}} \right)\hat{z}=m_{x}\hat{x}+m_{y}\hat{y}+m_{z}\hat{z}$$

(S1)

where $\theta$ and $\phi$ are the polar and azimuthal angles of the DW magnetization with respect to the $\hat{u}$ and $\hat{p}$ directions, respectively, at $\left( x,y,z \right)$ (see Fig. S3a).

With metric vectors $\vec{g}_{p,r}$, we obtain metric tensor $g_{\mu\nu}\equiv\vec{g}_{\mu}\cdot\vec{g}_{\nu}$ where $\mu,\nu=p,r$ as follows: $\left( g_{\mu\nu} \right)=\left( \begin{matrix} 1+\xi^{2}r^{2} & 0 \\ 0 & 1 \end{matrix} \right)$ in which the basis vectors are $\left\{ \hat{p},\hat{r} \right\}$. Spin connection vector $\Omega_{\mu}\equiv\hat{p}\cdot\frac{\partial\hat{r}}{\partial\mu}$ ($\mu=p,r)$ becomes $\vec{\Omega}=\frac{1}{\sqrt{1+\xi^{2}r^{2}}}\left( \xi^{2}r,0 \right)$ (the basis vectors are $\left\{ \hat{p},\hat{r} \right\}$), while the second fundamental form $b_{\mu\nu}\equiv\hat{u}\cdot\frac{\partial\vec{g}_{\mu}}{\partial\nu}$ ($\mu,\nu=p,r$) becomes $\left( b_{\mu\nu} \right)=\frac{\xi}{\sqrt{1+\xi^{2}r^{2}}}\left( \begin{matrix} 0 & 1 \\ 1 & 0 \end{matrix} \right)$. This allows us to obtain the Hessian matrix: $\left( H_{\mu\nu} \right)\equiv\frac{\left( b_{\mu\nu} \right)}{\sqrt{g_{\mu\mu}g_{\nu\nu}}}=\frac{\xi}{1+\xi^{2}r^{2}}\left( \begin{matrix} 0 & 1 \\ 1 & 0 \end{matrix} \right)$. Then, we have the vector $\vec{\Gamma}\equiv\left[ \left( H_{\mu\nu} \right)\left( \begin{aligned} \cos\phi\\ \sin\phi\end{aligned} \right) \right]^{T}=\frac{\xi}{1+\xi^{2}r^{2}}\left( \sin\phi,\cos\phi\right)$ (the basis vectors are $\left\{ \hat{p},\hat{r} \right\}$).

The exchange energy per unit volume is given by

$\epsilon_{ex}=A_{ex}\left( \nabla\hat{m} \right)^{2}=A_{ex}\sum_{X=x,y,z} \left( \frac{\partial m_{X}}{\partial X} \right)^{2}$ (S2)

where $A_{ex}$ is the exchange stiffness constant. Note that the exchange interaction energy is determined by the local variation of magnetizations at each point on the ribbon surface. When the ribbon thickness is much smaller than width and length, $\epsilon_{ex}$ in twisted ribbon can be derived from the following equation^1^:

$$\epsilon_{ex}=A_{ex}\left[ \nabla\theta-\vec{\Gamma}\left( \phi\right) \right]^{2}+A_{ex}\left[ \sin\theta\left( \nabla\phi-\vec{\Omega} \right)-\cos\theta\frac{\partial\vec{\Gamma}\left( \phi\right)}{\partial\phi} \right]^{2}$$

$$=A_{ex}\left[ \left( \frac{1}{\sqrt{g_{pp}}}\frac{\partial\theta}{\partial p},\frac{1}{\sqrt{g_{rr}}}\frac{\partial\theta}{\partial r} \right)-\frac{\xi}{1+\xi^{2}r^{2}}\left( \sin\phi,\cos\phi\right) \right]^{2}+A_{ex}\left[ -\frac{\sin\theta}{\sqrt{1+\xi^{2}r^{2}}}\left( \xi^{2}r,0 \right)-\frac{\xi\cos\theta}{1+\xi^{2}r^{2}}\left( \cos\phi,-\sin\phi\right) \right]^{2}$$

$$=A_{ex}\left[ \frac{1}{1+\xi^{2}r^{2}}\left( \frac{\partial\theta}{\partial p} \right)^{2}+\left( \frac{\partial\theta}{\partial r} \right)^{2}-\frac{2\xi\sin\phi}{\left( 1+\xi^{2}r^{2} \right)^{\frac{3}{2}}}\frac{\partial\theta}{\partial p}-\frac{2\xi\cos\phi}{1+\xi^{2}r^{2}}\frac{\partial\theta}{\partial r}+\frac{\xi^{2}\left( 1+\cos^{2} \theta\right)}{\left( 1+\xi^{2}r^{2} \right)^{2}}+\frac{\xi^{4}r^{2}\sin^{2} \theta}{1+\xi^{2}r^{2}}+\frac{\xi^{3}r\sin2\theta\cos\phi}{\left( 1+\xi^{2}r^{2} \right)^{\frac{3}{2}}} \right]$$

$$=A_{ex}\left[ \frac{1}{1+\xi^{2}r^{2}}\left( \frac{\partial\theta}{\partial p} \right)^{2}+\left( \frac{\partial\theta}{\partial r} \right)^{2}-\frac{2\xi\sin\phi}{\left( 1+\xi^{2}r^{2} \right)^{\frac{3}{2}}}\frac{\partial\theta}{\partial p}-\frac{2\xi\cos\phi}{1+\xi^{2}r^{2}}\frac{\partial\theta}{\partial r}+\xi^{2}\left\{ \frac{2-\sin^{2} \theta}{\left( 1+\xi^{2}r^{2} \right)^{2}}+\frac{\xi^{2}r^{2}\sin^{2} \theta}{1+\xi^{2}r^{2}}+\frac{\xi r\sin2\theta\cos\phi}{\left( 1+\xi^{2}r^{2} \right)^{\frac{3}{2}}} \right\} \right]$$

(S3)

Here $\nabla\theta=\left( \frac{1}{\sqrt{g_{pp}}}\frac{\partial\theta}{\partial p},\frac{1}{\sqrt{g_{rr}}}\frac{\partial\theta}{\partial r} \right)=\left( \frac{1}{\sqrt{1+\xi^{2}r^{2}}}\frac{\partial\theta}{\partial p},\frac{\partial\theta}{\partial r} \right)$ and $\nabla\phi=\left( \frac{1}{\sqrt{g_{pp}}}\frac{\partial\phi}{\partial p},\frac{1}{\sqrt{g_{rr}}}\frac{\partial\phi}{\partial r} \right)$. Since $\phi$ is assumed to be uniform in the $q-\phi$ model here $\nabla\phi=\left( 0,0 \right).$ Using $r\xi\ll1$, (S3) can be approximated to be

$$\epsilon_{ex}\approx A_{ex}\left[ \left( \frac{\partial\theta}{\partial p} \right)^{2}+\left( \frac{\partial\theta}{\partial r} \right)^{2}-2\xi\frac{\partial\theta}{\partial p}\sin\phi-2\xi\frac{\partial\theta}{\partial r}\cos\phi-\xi^{2}\sin^{2} \theta\right]$$

(S4)

The local energy density per unit volume, $\epsilon$, except magnetostatic interactions, is given by

$\epsilon=\epsilon_{ex}+\epsilon_{an}=A_{ex}\left( \nabla\hat{m} \right)^{2}+K\sin^{2} \theta$

$$\epsilon\cong A_{ex}\left[ \left( \frac{\partial\theta}{\partial p} \right)^{2}+\left( \frac{\partial\theta}{\partial r} \right)^{2}-2\xi\frac{\partial\theta}{\partial p}\sin\phi-2\xi\frac{\partial\theta}{\partial r}\cos\phi\right]+\left( K-\xi^{2}A_{ex} \right)\sin^{2} \theta$$

(S5)

$A_{ex}\left[ \left( \frac{\partial\theta}{\partial p} \right)^{2}+\left( \frac{\partial\theta}{\partial r} \right)^{2} \right]$ corresponds to conventional local exchange energy density. Now it is clear that the twisting induced local exchange energy density, $\epsilon_{tor}=\epsilon_{tor}^{r}+\epsilon_{tor}^{p}=-2\xi A_{ex}\left[ \frac{\partial\theta}{\partial p}\sin\phi+\frac{\partial\theta}{\partial r}\cos\phi\right]$ (here $\epsilon_{tor}^{r}=-2\xi A_{ex}\frac{\partial\theta}{\partial p}\sin\phi$ and $\epsilon_{tor}^{p}=-2\xi A_{ex}\frac{\partial\theta}{\partial r}\cos\phi$), is an effective DMI energy density induced by the geometrical twisting while $-\xi^{2}A_{ex}$ corresponds to an effective anisotropy induced by the twisting effect. It is the $\mathcal{E}_{tor}$ that is responsible for the twisting chirality dependent DW motion, which will be discussed in the end of this section. Most importantly, $\epsilon_{tor}^{r}$ and $\epsilon_{tor}^{p}$ induce effective DMI torsional fields $\vec{H}_{tor}^{r}$ and $\vec{H}_{tor}^{p}$, respectively, that are collinear with $\hat{r}$ and $\hat{p}$, thereby favoring chiral Bloch-type and chiral Néel-type walls. Note that torsional field is a DMI field as typically discussed in curvilinear magnetism. Consequently, the DW energy $\mathcal{E}$ (per unit thickness) on a twist ribbon can be obtained: $\mathcal{E=}\int\epsilon dpdr$.

By taking functional derivatives and functional variational method, i.e, $\frac{\delta\mathcal{E}}{\delta\theta}=\int\left( \frac{\partial\epsilon}{\partial\theta}-\nabla\cdot\frac{\partial\epsilon}{\partial\nabla\theta} \right)dpdr=0$ and $\frac{\delta\mathcal{E}}{\delta\phi}=\int\left( \frac{\partial\epsilon}{\partial\phi}-\nabla\cdot\frac{\partial\epsilon}{\partial\nabla\phi} \right)dpdr=0$, the domain wall profile can be derived:

$$\frac{\partial\epsilon}{\partial\theta}-\frac{\partial}{\partial p}\frac{\partial\epsilon}{\partial\left( \frac{\partial\theta}{\partial p} \right)}-\frac{\partial}{\partial r}\frac{\partial\epsilon}{\partial\left( \frac{\partial\theta}{\partial r} \right)}=0$$

$$\frac{\partial\epsilon}{\partial\phi}-\frac{\partial}{\partial p}\frac{\partial\epsilon}{\partial\left( \frac{\partial\phi}{\partial p} \right)}-\frac{\partial}{\partial r}\frac{\partial\epsilon}{\partial\left( \frac{\partial\phi}{\partial r} \right)}=0$$

(S6a,b)

In the $q-\phi$ model, a trivial solution $\phi\left( p,r,t \right)=\psi\left( t \right)$ to eq. (S6b) is taken for simplicity so that $\psi\left( t \right)$ is spatially uniform in $q-\phi$ model. Note, however, that $\psi$ has $t$-dependence, i.e. $\psi\left( t \right)$. Hence, we obtain the following from the eqs. (S5) and (S6):

$$2\frac{\partial^{2}\theta}{\partial p^{2}}+2\frac{\partial^{2}\theta}{\partial r^{2}}-\left( \frac{K}{A_{ex}}-\xi^{2} \right)\sin2\theta=0$$

$\phi=\psi$ (uniform)

(S7a,b)

Since $A_{ex}=\sim34$ pJ/m and $K=\sim5.2\times{10}^{6}$ erg/cm^3^ in our devices, $\frac{K}{A_{ex}}\gg\xi^{2}$ and, consequently, the eq. (S7a) can be approximated as

$$2\frac{\partial^{2}\theta}{\partial p^{2}}+2\frac{\partial^{2}\theta}{\partial r^{2}}-\frac{K}{A_{ex}}\sin2\theta=0$$

(S8)

The simplest non-trivial solution to eq. (S8) is $\theta=2\arctan\exp\left[ \pm\frac{a_{1}p+a_{2}r}{\Delta} \right]$ with a constraint: $a_{1}^{2}+a_{2}^{2}=1$, from which $\chi$ can be defined such that $a_{1}=\cos\chi$ and $a_{2}=\sin\chi$. Thus, the new degree of freedom is “$\chi$” that corresponds to the DW tilting. Here $\Delta=\sqrt{\frac{A_{ex}}{K}}$ is the DW’s width parameter (see Fig. S4). The DW width is $\pi\Delta$. Note that there is a conjugate of $\phi$, that is, $q$, that is another degree of freedom in the DW profile and eq. (S8). $q$ corresponds to the DW middle position along $p$ coordinate. Note that $q$ and $\psi$ do not have coordinate-dependence but time-dependence only, that is, $q\left( t \right)$ and $\psi\left( t \right)$. By plugging $q$ into the DW profile function, we finally obtain the DW profile function on twisted ribbon surface,

$\theta=2\arctan\exp\left[ \pm\frac{\left( p-q \right)\cos\chi+r\sin\chi}{\Delta} \right]$ (S9)

where $+$ and $-$ correspond to $⨀\bigotimes$ and$\bigotimes⨀$ configurations, respectively. Various cases of DW configurations and twisting chiralities are illustrated in Fig. S5. Using (S9) and the approximation, the eq. (S4) becomes

$$\epsilon\cong A_{ex}\left[ \left( \frac{\partial\theta}{\partial p} \right)^{2}+\left( \frac{\partial\theta}{\partial r} \right)^{2}-2\xi\frac{\partial\theta}{\partial p}\sin\psi-2\xi\frac{\partial\theta}{\partial r}\cos\psi\right]$$

$$=\left( \frac{A_{ex}}{\Delta^{2}}+K \right)\sin^{2} \theta\mp\frac{2\xi A_{ex}}{\Delta}\sin\theta\sin\left( \psi+\chi\right)$$

(S10)

Here $\frac{\partial\theta}{\partial p}=\pm\frac{\sin\theta}{\Delta}\cos\chi$ and $\frac{\partial\theta}{\partial r}=\pm\frac{\sin\theta}{\Delta}\sin\chi$ are used (the upper and lower signs correspond to $⨀\bigotimes$ and$\bigotimes⨀$ configurations, respectively).


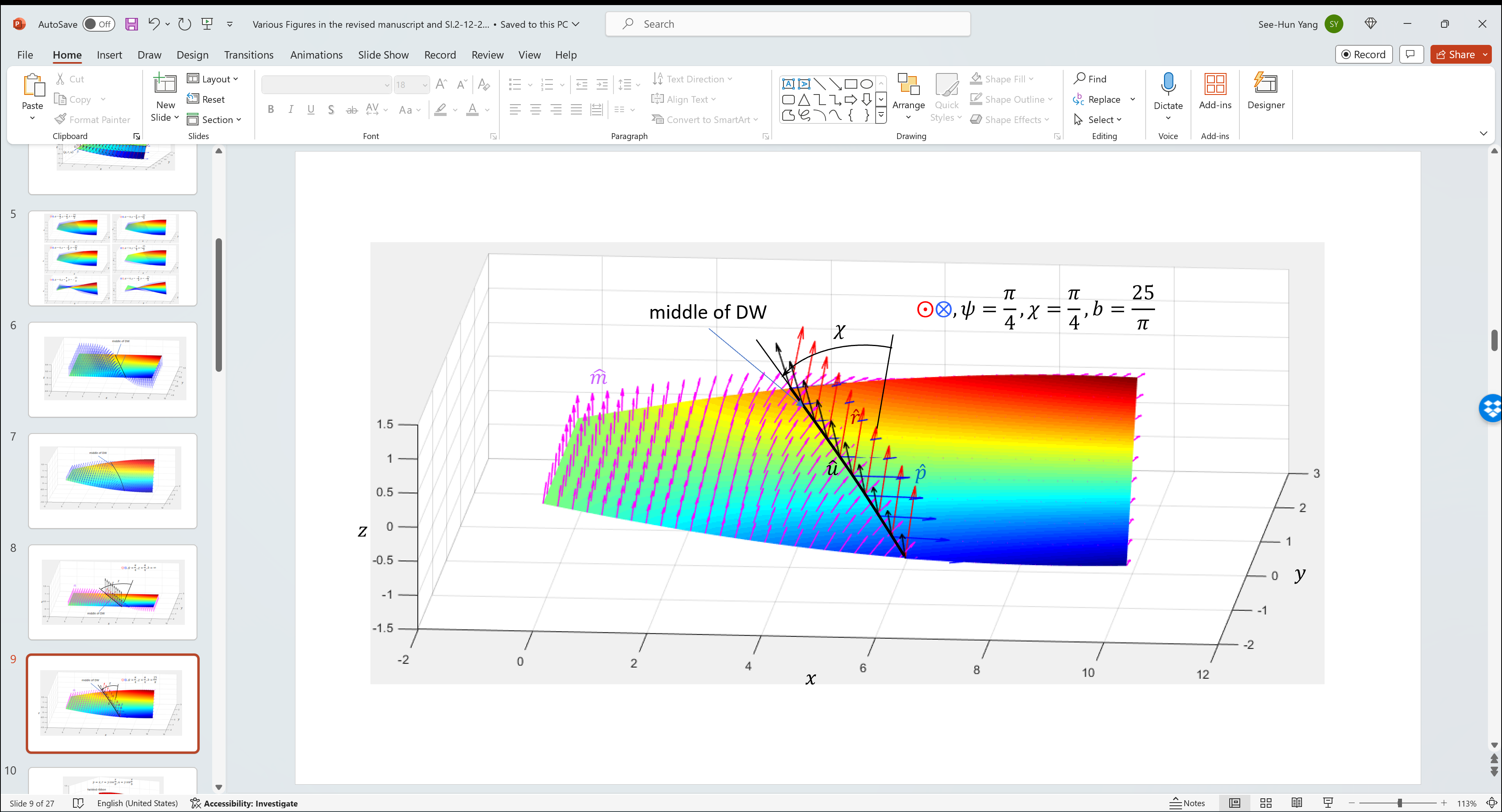


**Figure S4 |** Calculated illustration of magnetizations (magenta) of domain and $⨀\bigotimes$ DW with $q=5$, $L=10$, $w=2.5$, $\psi=\frac{\pi}{4}$, $\chi=\frac{\pi}{4}$, and $b=25/\pi$. Orthogonal unit vectors $\left\{ \hat{p},\hat{r},\hat{u} \right\}$ are displayed at the middle of DW.


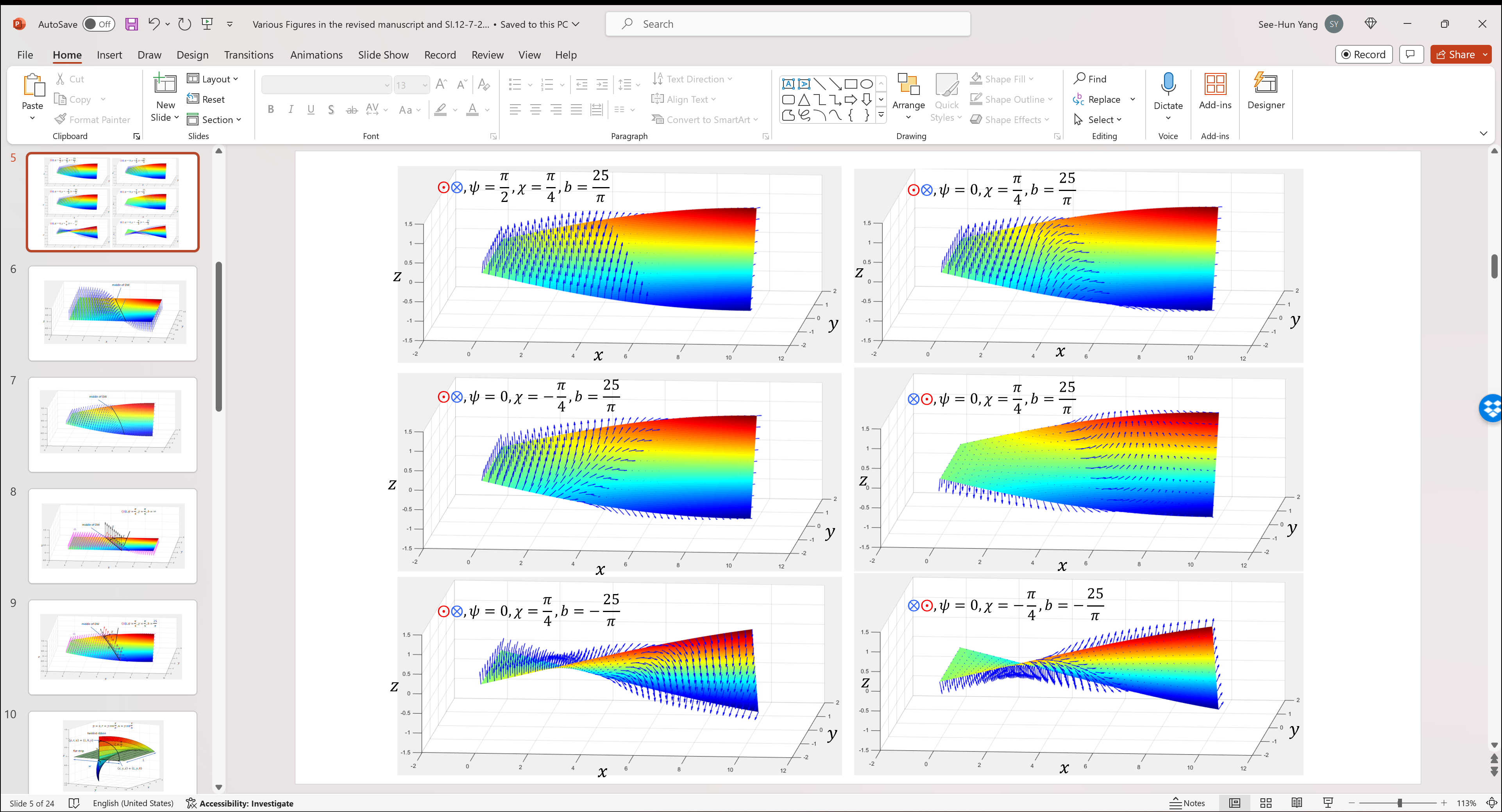


**a**

**b**

**cc**

**d**

**e**

**f**

**Figure S5** | Calculated illustration of various cases of DWs on 3D twisted ribbons based on eqs. (S1) and (S9) with $w=2$, $q=5$, and $L=10$.

Most importantly, it is the second term, $\mp\frac{2\xi A_{ex}}{\Delta}\sin\theta\sin\left( \psi+\chi\right)$, that gives rise to the twisting driven DW motion as discussed below.

The Lagrangian $\mathcal{L}$ and non-conservative $\mathcal{F}$ that include magnetostatic interaction energy per unit area $\epsilon_{ms}$ are given by

$$\mathcal{L=}\int_{-\frac{w}{2}}^{\frac{w}{2}} \int_{0}^{L} \left[ \left( \frac{A_{ex}}{\Delta^{2}}+K \right)\sin^{2} \theta\mp\frac{2\xi A_{ex}}{\Delta}\sin\theta\sin\left( \psi+\chi\right)+\epsilon_{ms}\left( q,\theta, \psi,\chi\right)-M_{s}H_{p}\sin\theta\cos\left( \psi-\psi_{H} \right)-M_{s}H_{DM}\sin\theta\cos\left( \psi-\chi\right)+\frac{M_{s}}{\gamma}\psi\dot{\theta}\sin\theta\right]\sqrt{g}dpdr$$

$$=2w\sec\chi\left( \frac{A_{ex}}{\Delta}+K\Delta\right)\mp2\pi w\xi A_{ex}\sin\left( \psi+\chi\right)\sec\chi-\pi\Delta wM_{s}H_{p}\sec\chi\cos\left( \psi-\psi_{H} \right)-\pi\Delta wM_{s}H_{DM}\sec\chi\cos\left( \psi-\chi\right)\mp\frac{M_{s}}{\gamma}2w\dot{q}\psi+\mathcal{E}_{ms}\left( q,\psi,\chi\right)$$

(S11)

$$\mathcal{F=}\int_{-\frac{w}{2}}^{\frac{w}{2}} \int_{0}^{L} \left[ {\dot{\hat{m}}}^{2}+\frac{2\gamma}{\alpha}H_{SH}\hat{m}\times\hat{r}\cdot\dot{\hat{m}} \right]dxdy\approx\int_{-\frac{w}{2}}^{\frac{w}{2}} \int_{0}^{L} \left[ \dot{\theta}^{2}+\dot{\psi}^{2}\sin^{2} \theta-\frac{2\gamma}{\alpha}H_{SH}\dot{\theta}\cos\psi+\frac{2\gamma}{\alpha}H_{SH}\dot{\psi}\sin2\theta\sin\psi\right]\sqrt{g}dpdr$$

$$=\frac{\alpha M_{s}}{2\gamma}\left[ \frac{2\dot{q^{2}}}{\Delta}w\cos\chi+\frac{w}{6\Delta}\dot{\chi}^{2}\csc^{3} \chi\left( w^{2}+\pi^{2}\Delta^{2}\cos^{2} \chi\right) \right]\pm\pi M_{s}H_{SH}\dot{q}\cos\psi+\frac{\alpha M_{s}w\Delta}{\gamma}\dot{\psi}^{2}\sec\chi$$

(S12)

where the upper and lower signs correspond to $⨀\bigotimes$ and $\bigotimes⨀$ configurations, respectively. Here $\gamma$ is the gyromagnetic ratio, $\alpha$ is Gilbert damping, and $H_{SH}$ is the spin Hall parameter: $H_{SH}=\frac{h\theta_{SH}J}{2eMt_{FM}}$ that measures the magnitude of spin current induced by the spin Hall effect in the Pt layer. $h, \theta_{SH}, e$and $t_{FM}$ denote the Planck constant, spin Hall angle, electron charge and ferromagnetic layer thickness, respectively. $H_{p}$ is the external in-plane field, $\psi_{H}$ is the angle of $H_{p}$ with respect to $\hat{p}$ in the $\hat{p}-\hat{r}$ local plane. $H_{DM}$ is the DM field that is defined by $H_{DM}=\frac{D}{M_{s}\Delta}$ where $D$ is the DMI constant. Note that $g=\det\left( g_{\mu\nu} \right)=1+\xi^{2}r^{2}\approx1$ is used here and onwards. The magnetostatic interaction energy $\mathcal{E}_{ms}=\int\epsilon_{ms}\sqrt{g}dpdr$.

.Finally, the equations of motion can be derived from eqs. (S11) and (S12) using Lagrange-Rayleigh formalism $\frac{\partial\mathcal{L}}{\partial\beta}-\frac{d}{dt}\frac{\partial\mathcal{L}}{\partial\dot{\beta}}+\frac{\partial\mathcal{F}}{\partial\dot{\beta}}=0$ where $\beta=q, \psi$ and $\chi$:

$$\dot{q}=\frac{\gamma\sec\chi}{1+\alpha^{2}}\left\{ -\frac{\pi{\xi A}_{ex}}{M_{s}}\cos\left( \psi+\chi\right)\pm\frac{\pi}{2}\Delta H_{p}\sin\left( \psi-\psi_{H} \right)\pm\frac{\pi}{2}\Delta H_{DM}\sin\left( \psi-\psi_{DM} \right)\mp\frac{\pi}{2}\alpha\Delta H_{SH}\cos\psi\pm\frac{1}{2wM_{s}}\left[ \frac{\partialℇ_{ms}\left( q,\psi,\chi\right)}{\partial\psi}\cos\chi\mp\alpha\Delta\frac{\partial\mathcal{E}_{ms}\left( q,\psi,\chi\right)}{\partial q} \right] \right\}$$

$$\dot{\psi}=\frac{\gamma}{1+\alpha^{2}}\left\{ \pm\frac{\pi{\alpha\xi A}_{ex}}{M_{s}\Delta}\cos\left( \psi+\chi\right)-\frac{\pi}{2}\alpha\Delta H_{p}\sin\left( \psi-\psi_{H} \right)-\frac{\pi}{2}\alpha\Delta H_{DM}\sin\left( \psi-\psi_{DM} \right)-\frac{\pi}{2}\Delta H_{SH}\cos\psi-\frac{1}{2w{\Delta M}_{s}}\left[ \alpha\frac{\partialℇ_{ms}\left( q,\psi,\chi\right)}{\partial\psi}\cos\chi\pm\Delta\frac{\partial\mathcal{E}_{ms}\left( q,\psi,\chi\right)}{\partial q} \right] \right\}$$

$$\dot{\chi}=\frac{-\frac{\sigma}{M_{s}\Delta}\tan\chi+\pi H_{DM}\sin\left( \psi-\chi\right)+\frac{\pi\xi A_{ex}}{2M_{s}\Delta}\cos\psi\sec\chi-\frac{1}{wM_{s}\Delta}\frac{\partial\mathcal{E}_{dip}\left( q,\psi,\chi\right)}{\partial\chi}\cos\chi}{\frac{\pi^{2}\alpha}{6\gamma}\left[ \left( \frac{w}{\pi\Delta} \right)^{2}\sec^{2} \chi+\tan^{2} \chi\right]}$$

(S13a,b,c)

where the upper and lower signs correspond to $⨀\bigotimes$ and $\bigotimes⨀$, respectively, and $\sigma=4\sqrt{A_{ex}K}-\pi\Delta M_{s}H_{p}\cos\left( \psi-\psi_{H} \right)-\pi\Delta M_{s}H_{DM}\cos\left( \psi-\chi\right).$ An extrinsic periodic pinning potential $V_{pin}=V_{0}\sin\frac{2\pi\left( q-q_{shift} \right)}{q_{0}}$ that may be caused by ribbon edges and inhomogeneous films^3,4^ has been added to describe a finite $J_{c}$ (Fig. 3).

Now let us discuss how the interaction of twisting chirality with the exchange interaction affects the current driven chiral DW motions from $\mathcal{E}_{tor}=\int\epsilon_{tor}\sqrt{g}dpdr=$ $\mathcal{E}_{tor}^{r}+\mathcal{E}_{tor}^{p}$, that is geometrical twisting induced magnetic energy per unit thickness. Let us first investigate $\mathcal{E}_{tor}^{r}=\int\epsilon_{tor}^{r}\sqrt{g}dpdr=\mp2\pi w{\xi A}_{ex}\sin\psi$ (upper sign: $⨀\bigotimes$, lower sign: $\bigotimes⨀$). Note that $\mathcal{E}_{tor}^{r}$ has no dependence on $\chi$. $\frac{\partial\theta}{\partial p}>0$and $\frac{\partial\theta}{\partial p}<0$ correspond to $⨀\bigotimes$ ($\theta$: $0\to\pi$) and $\bigotimes⨀$ ($\theta:$ $\pi\to0$) configuration, respectively. $\mathcal{E}_{tor}^{r}$ results in that, for $⨀\bigotimes$ and $\xi>0$ (right-handed twist), $\psi=\frac{\pi}{2}$ (left-handed Bloch-type wall: the lowest energy state) is favored against $\psi=0,\pi$ (Néel-type walls: the intermediate energy state) and $\frac{3\pi}{2}$ (right-handed Bloch-type wall: the highest energy state). Consequently, it is obvious that the effective torsional field $\vec{H}_{tor}^{r}=-\frac{\delta\mathcal{E}_{tor}^{r}}{\delta\vec{M}}$ forms maxima at $\psi=0,\pi$, and minima at $\psi=\frac{\pi}{2},\frac{3\pi}{2}$, thereby leading to $\vec{H}_{tor}^{p}$ along $+\hat{r}$-direction ($\psi=\frac{3\pi}{2}\to\frac{\pi}{2}$). This is because $H_{tor}^{r}\propto-\frac{{\partial\mathcal{E}}_{tor}^{r}}{\partial\psi}\propto\pm cos \psi$. The direction of torsional torque $\vec{\tau}_{tor}^{r}=-\gamma\vec{m}\times\vec{H}_{tor}^{r}=\mp\pi\gamma\xi A_{ex}\cos\psi\hat{u}$ ($\gamma$: the gyromagnetic ratio, upper sign: $⨀ \leftarrow\bigotimes$ , lower sign: $\bigotimes\to⨀$) is determined by the orientation of $\vec{m}$, that is, $\psi$. Note that $\psi$ is determined by the SOT and the interface induced DMI field $\vec{H}_{DM}$ that favors chiral Néel-type walls. For left-handed chirality ($⨀ \leftarrow\bigotimes$ and $\bigotimes\to⨀$), $\tau_{tor}^{r}$ is along $+\hat{u}$-direction for both $⨀\bigotimes$ and $\bigotimes⨀$, since $\cos\psi<0$ ($⨀\bigotimes$) and $\cos\psi>0$ ($\bigotimes⨀$). Consequently, the motion of $⨀\bigotimes$ DW gets boosted by $\vec{\tau}_{tor}^{r}$ whilst of $⨀\bigotimes$ DW velocity is compensated by $\vec{\tau}_{tor}^{r}$ as experimentally observed.

Next, let us investigate $\mathcal{E}_{tor}^{p}=\int\epsilon_{tor}^{p}\sqrt{g}dpdr=\mp2\pi\gamma\xi A_{ex}\cos\psi\tan\chi$. Another local geometrical twisting exchange energy per thickness, $\mathcal{E}_{tor}^{p}$, is induced when $\chi\neq0$. This is because DW is formed along not only $\hat{p}$ but $\hat{r}$ axes, and the ribbon is twisted around the $\hat{r}$ axis as well when $\chi\neq0$. Note that $\frac{\partial\theta}{\partial r}=\pm\frac{\sin\theta}{\Delta}\sin\chi$ in eq. (S10) (upper sign: $⨀\bigotimes$, lower sign: $\bigotimes⨀$). We have $\frac{\partial\theta}{\partial r}>0$for $⨀\bigotimes$ with $\chi>0$ or $\bigotimes⨀$ with $\chi<0$, while $\frac{\partial\theta}{\partial r}<0$for $⨀\bigotimes$ with $\chi<0$ or $\bigotimes⨀$ with $\chi>0$. Consequently, $\mathcal{E}_{tor}^{p}$ varies depending on $b$, $\psi$, and $\chi$. Note that $\mathcal{E}_{tor}^{p}$ favors chiral Néel-type walls like iDMI while $\mathcal{E}_{tor}^{r}$ favors chiral Bloch-type walls.

When $\xi>0$ (right-handed), $\mathcal{E}_{tor}^{p}$ dictates that for $⨀\bigotimes$ with $\chi<0$ or $\bigotimes⨀$ with $\chi>0$, $\psi=\pi$ (left-handed $⨀\leftarrow\bigotimes$ or right-handed $\bigotimes\leftarrow⨀$ Néel-type wall) are favored against $\psi=\frac{\pi}{2},\frac{3\pi}{2}$ (Bloch-type walls). $\mathcal{E}_{tor}^{p}$-induced effective fields $\vec{H}_{tor}^{p}=-\frac{\delta\mathcal{E}_{tor}^{p}}{\delta\vec{M}}$ point along $-\hat{p}$-direction ($\psi=0\to\pi$). On the other hand, for $⨀\bigotimes$ with $\chi>0$ or $\bigotimes⨀$ with $\chi<0$, $\psi=0$ (right-handed $⨀\to\bigotimes$ or left-handed $\bigotimes\to⨀$ Néel-type wall) are favored against $\psi=\frac{\pi}{2},\frac{3\pi}{2}$. $\mathcal{E}_{tor}^{p}$-induced effective fields $\vec{H}_{tor}^{p}=-\frac{\delta\mathcal{E}_{tor}^{p}}{\delta\vec{M}}$ point along $+\hat{p}$-direction ($\psi=\pi\to0$). The direction of $\vec{H}_{tor}^{p}$-induced torque $\vec{\tau}_{tor}^{p}=-\gamma\vec{m}\times\vec{H}_{tor}^{p}$ is determined by $\chi$ and the relative angle between $\vec{m}$ and spin Hall current induced spin polarization $\vec{S}$. If the spin polarization $\vec{S}$ is along $-\hat{r}$ that corresponds to the current along $+\hat{p}$-direction in Pt, $\vec{\tau}_{tor}^{p}=\pm\pi\gamma\xi A_{ex}\cos\psi\tan\chi\hat{u}$ is along $+\hat{u}$-direction so that the motion of $⨀\bigotimes$ DW with $\chi<0$ is enhanced by $\vec{\tau}_{tor}^{p}$ while the motion of $\bigotimes⨀$ DW with $\chi>0$ is reduced. In contrast for $⨀\bigotimes$ with $\chi>0$ or $\bigotimes⨀$ with $\chi<0$, $\vec{\tau}_{tor}^{p}$ is oriented along $-\hat{u}$-direction so that the $⨀\bigotimes$ DW velocity is reduced by $\vec{\tau}_{tor}^{p}$ while the $\bigotimes⨀$ DW velocity is increased. When $b<0$ (left-handed twist), vice versa.

Finally, when $\tau_{tor}^{r}$ and $\tau_{tor}^{p}$ are combined together, the total torsional torque $\vec{\tau}_{tor}=\vec{\tau}_{tor}^{r}+\vec{\tau}_{tor}^{p}$=$\mp\pi\gamma\xi A_{ex}\cos\left( \psi+\chi\right)\sec\chi\hat{u}$. Such torsional fields and torques originate from the interplay of exchange with curvilinear geometry: the exchange energy is large (small) when the DW magnetization rotates in the same (opposite) direction as the ribbon twists around. A comprehensive summary is presented in the Extended Data Tables.

**Supplementary Note 3: Magnetostatic interaction in chiral domain walls on 3D twisted ribbons**

We investigate the effect of magnetostatic interaction in chiral domain walls on 3D twisted ribbons. Magnetostatic interactions in magnetic systems are long-range interactions that include not only local but nonlocal ones. Especially, the nonlocal effects in magnetostatic interactions have been recently recognized to play a critical role in curvilinear magnetic systems. The magnetostatic energy $\mathcal{E}_{ms}$ per unit thickness is given by^5,6^

$\mathcal{E}_{ms}=\mathcal{E}_{ms}^{S-S}+\mathcal{E}_{ms}^{B-B}+\mathcal{E}_{ms}^{B-S}$ (S14)

$\mathcal{E}_{ms}^{S-S}$, $\mathcal{E}_{ms}^{B-B}$ and $\mathcal{E}_{ms}^{B-S}$ correspond to magnetostatic energies induced by interactions between surface charges at different surfaces per length, bulk-bulk charges per thickness, and bulk-surface charges for all surfaces per thickness, respectively, that are given by

$$\mathcal{E}_{ms}^{S-S}=\frac{M_{s}^{2}}{2L}\iint\frac{\left( \hat{m}\cdot\hat{n} \right)\left( \hat{m}^{'}\cdot\hat{n}^{'} \right)}{\left| \vec{r}-\vec{r}^{'} \right|}dS^{'}dS$$

$$\mathcal{E}_{ms}^{B-B}=\frac{M_{s}^{2}}{2t}\iint\frac{\left( \nabla\cdot\hat{m} \right)\left( \nabla^{'}\cdot\hat{m}^{'} \right)}{\left| \vec{r}-\vec{r}^{'} \right|}dV^{'}dV$$

$$\mathcal{E}_{ms}^{B-S}=\frac{M_{s}^{2}}{2t}\iint\frac{\left( \nabla\cdot\hat{m} \right)\left( \hat{m}^{'}\cdot\hat{n}^{'} \right)}{\left| \vec{r}-\vec{r}^{'} \right|}dS'dV$$

(S15a,b,c)

In our 3D twisted ribbons, since we have

$\hat{m}\cdot\hat{n}=\cos\theta$,

$$\nabla\cdot\hat{m}\cong\pm\frac{\sin2\theta}{2\Delta}\left[ \cos\left( \psi-\chi\right)+\xi r \right]$$

in which $\xi r\ll1$ is used, the following can be readily derived to be

$$\mathcal{E}_{ms}^{S-S}=\frac{M_{s}^{2}}{2L}R\left( q,\chi\right)$$

$$\mathcal{E}_{ms}^{B-B}=\frac{M_{s}^{2}}{2t}\left[ U\left( q,\chi\right)\cos^{2} \left( \psi-\chi\right)\sec\chi+\xi^{2}V\left( q,\chi\right) \right].$$

(S16a,b)

Here $R\left( q,\chi\right)=\iint\frac{\cos\theta\cos\theta^{'}}{\sqrt{\left( x-x^{'} \right)^{2}+\left( y-y^{'} \right)^{2}+\left( z-z^{'} \right)^{2}}}\sqrt{g'g}dp^{'}dr^{'}dpdr$

$=\int_{-\frac{w}{2}}^{\frac{w}{2}} \int_{0}^{L} \int_{-\frac{w}{2}}^{\frac{w}{2}} \int_{0}^{L} \frac{\tanh\left[ \pm\frac{\left( p-q \right)\cos\chi+r\sin\chi}{\Delta} \right]\tanh\left[ \pm\frac{\left( p^{'}-q \right)\cos\chi+r^{'}\sin\chi}{\Delta} \right]}{\sqrt{\left( p-p^{'} \right)^{2}+\left( r\cos\xi p-r^{'}\cos{\xi p}^{'} \right)^{2}+\left( r\sin\xi p+t-r^{'}\sin{\xi p}^{'} \right)^{2}}}\sqrt{g'g}dp^{'}dr^{'}dpdr$,

$U\left( q,\chi\right)=\frac{t^{2}\cos\chi}{4\Delta^{2}}\iint\frac{\sin2\theta\sin2\theta^{'}}{\sqrt{\left( x-x^{'} \right)^{2}+\left( y-y^{'} \right)^{2}+\left( z-z^{'} \right)^{2}}}\sqrt{g'g}dp^{'}dr^{'}dpdr$

$\approx\frac{t^{2}\cos\chi}{\Delta^{2}}\int_{-\frac{w}{2}}^{\frac{w}{2}} \int_{0}^{L} \int_{-\frac{w}{2}}^{\frac{w}{2}} \int_{0}^{L} \frac{\left\{ \begin{aligned} \tanh\left[ \pm\frac{\left( p-q \right)\cos\chi+r\sin\chi}{\Delta} \right]\tanh\left[ \pm\frac{\left( p^{'}-q \right)\cos\chi+r^{'}\sin\chi}{\Delta} \right]\times\\ \mathrm{sech} \left[ \pm\frac{\left( p-q \right)\cos\chi+r\sin\chi}{\Delta} \right]\mathrm{sech} \left[ \pm\frac{\left( p^{'}-q \right)\cos\chi+r^{'}\sin\chi}{\Delta} \right] \end{aligned} \right\}}{\sqrt{\left( p-p^{'} \right)^{2}+\left( r\cos\xi p-r^{'}\cos{\xi p}^{'} \right)^{2}+\left( r\sin\xi p-r^{'}\sin{\xi p}^{'} \right)^{2}}}\sqrt{g'g}dp^{'}dr^{'}dpdr$,

$V\left( q,\chi\right)=\frac{t^{2}}{4\Delta^{2}}\iint\frac{rr^{'}\sin2\theta\sin2\theta^{'}}{\sqrt{\left( r-r^{'} \right)^{2}+\left( y-y^{'} \right)^{2}+\left( z-z^{'} \right)^{2}}}\sqrt{g'g}dp^{'}dr^{'}dpdr$

$=\frac{t^{2}}{\Delta^{2}}\int_{-\frac{w}{2}}^{\frac{w}{2}} \int_{0}^{L} \int_{-\frac{w}{2}}^{\frac{w}{2}} \int_{0}^{L} \frac{rr^{'}\left\{ \begin{aligned} \tanh\left[ \pm\frac{\left( p-q \right)\cos\chi+r\sin\chi}{\Delta} \right]\tanh\left[ \pm\frac{\left( p^{'}-q \right)\cos\chi+r^{'}\sin\chi}{\Delta} \right]\times\\ \mathrm{sech} \left[ \pm\frac{\left( p-q \right)\cos\chi+r\sin\chi}{\Delta} \right]\mathrm{sech} \left[ \pm\frac{\left( p^{'}-q \right)\cos\chi+r^{'}\sin\chi}{\Delta} \right] \end{aligned} \right\}}{\sqrt{\left( p-p^{'} \right)^{2}+\left( r\cos\xi p-r^{'}\cos{\xi p}^{'} \right)^{2}+\left( r\sin\xi p-r^{'}\sin{\xi p}^{'} \right)^{2}}}\sqrt{g'g}dp^{'}dr^{'}dpdr$.

(S17a,b,c)

where upper and lower signs correspond to $⨀\bigotimes$ and $\bigotimes⨀$, respectively. Note that in eq. (15b) and (16b),

$$\frac{t^{2}}{2\Delta^{2}}\iint\frac{r\sin2\theta\sin2\theta^{'}}{\sqrt{\left( x-x^{'} \right)^{2}+\left( y-y^{'} \right)^{2}+\left( z-z^{'} \right)^{2}}}\sqrt{g'g}dp^{'}dr^{'}dpdr=$$

$=\frac{2t^{2}}{\Delta^{2}}\int_{-\frac{w}{2}}^{\frac{w}{2}} \int_{0}^{L} \int_{-\frac{w}{2}}^{\frac{w}{2}} \int_{0}^{L} \frac{y^{'}\left\{ \begin{aligned} \tanh\left[ \pm\frac{\left( p-q \right)\cos\chi+r\sin\chi}{\Delta} \right]\tanh\left[ \pm\frac{\left( p^{'}-q \right)\cos\chi+r^{'}\sin\chi}{\Delta} \right]\times\\ \mathrm{sech} \left[ \pm\frac{\left( p-q \right)\cos\chi+r\sin\chi}{\Delta} \right]\mathrm{sech} \left[ \pm\frac{\left( p^{'}-q \right)\cos\chi+r^{'}\sin\chi}{\Delta} \right] \end{aligned} \right\}}{\sqrt{\left( p-p^{'} \right)^{2}+\left( r\cos\xi p-r^{'}\cos{\xi p}^{'} \right)^{2}+\left( r\sin\xi p-r^{'}\sin{\xi p}^{'} \right)^{2}}}\sqrt{g'g}dp^{'}dr^{'}dpdr=0$,

$\mathcal{E}_{ms}^{B-B}$ has no dependence on the twisting chirality as shown in eqs. (S16b) and (S17b,c). Since our magnetic films on 3D twisted ribbons are thin (~ 1 nm thick), there are nearly identical magnetic charge distribution and magnitude with opposite signs for the top and bottom surfaces of 3D twisted ribbon. Consequently, eq. (S15c) shows that the interaction between top surface and bulk charges nearly compensates the one between bottom surface and bulk charges, thereby leading to $\mathcal{E}_{dip}^{B-S}=0.$ Finally, the total magnetostatic energy density is:

$$\mathcal{E}_{ms}=\frac{M_{s}^{2}}{2L}R\left( q,\chi\right)+\frac{M_{s}^{2}}{2t}\left[ U\left( q,\chi\right)\cos^{2} \left( \psi-\chi\right)\sec\chi+\xi^{2}V\left( q,\chi\right) \right]$$

(S18)

$R\left( q,\chi\right)$, $U\left( q,\chi\right)$ and $V\left( q,\chi\right)$ are numerically calculated. We find that $U\left( q,\chi\right)$ and $V\left( q,\chi\right)$ do not have $q$-dependence, i.e. $U\left( q,\chi\right)=U\left( \chi\right)$ and $V\left( q,\chi\right)=V\left( \chi\right)$ as shown in Fig. S6. In addition, $R\left( q,\chi\right)$, $U\left( \chi\right)$ and $U\left( \chi\right)$ have weak $\chi$-dependence so that $R\left( q,\chi\right)\sim R\left( q \right)$, $U\left( \chi\right)\sim U$ and $V\left( \chi\right)\sim V$ (see Fig. S6). Note that $\frac{M_{s}^{2}}{2t}R\left( q \right)$ and $\frac{M_{s}^{2}}{2t}U\cos^{2} \left( \psi-\chi\right)\sec\chi$ correspond to the demagnetization energies for magnetic thin films and DW shape anisotropy, respectively. To check this, assuming that the ribbon is very large and there is no DW in the ribbon, $\frac{M_{s}^{2}}{2L}R\left( q=\pm\infty\right)\sim2\pi M_{S}^{2}wL$ thus leading to $R\left( q \right)\sim4\pi wL^{2}\sim3.1\times{10}^{-15}$ m^3^. This value is similar with those for $q=2$ and $8$ μm in the numerical calculations shown in Fig. S6a. The DW shape anisotropy is the magnetostatic energy difference between Néel-type wall and Bloch-type wall. Typically, Bloch-type wall is favored by the DW shape anisotropy since the DW width is comparable with $t$ but much smaller than $w$. Since the DW shape anisotropy fields $H_{k}\sim4\pi M_{s}\frac{t}{t+\Delta} ADDIN ZOTERO\_ITEM CSL\_CITATION \{"citationID":"5pcoddbb","properties":\{"formattedCitation":"\backslash\backslash super 3\backslash\backslash nosupersub\{\}","plainCitation":"3","noteIndex":0\},"citationItems":[\{"id":480,"uris":["http://zotero.org/users/local/pl8qgtBf/items/B29KERZD"],"itemData":\{"id":480,"type":"article-journal","abstract":"Domain walls can be driven by current at very high speeds in nanowires formed from ultra-thin, perpendicularly magnetized cobalt layers and cobalt/nickel multilayers deposited on platinum underlayers due to a chiral spin torque. An important feature of this torque is a magnetic chiral exchange field that each domain wall senses and that can be measured by the applied magnetic field amplitude along the nanowire where the domain walls stop moving irrespective of the magnitude of the current. Here we show that this torque is manifested when the magnetic layer is interfaced with metals that display a large proximity-induced magnetization, including iridium, palladium and platinum but not gold. A correlation between the strength of the chiral spin torque and the proximity-induced magnetic moment is demonstrated by interface engineering using atomically thin dusting layers. High domain velocities are found where there are large proximity-induced magnetizations in the interfaced metal layers.","container-title":"Nature Communications","DOI":"10.1038/ncomms4910","ISSN":"2041-1723","language":"en","license":"2014 Nature Publishing Group","page":"3910","source":"www.nature.com","title":"Chiral spin torque arising from proximity-induced magnetization","volume":"5","author":[\{"family":"Ryu","given":"Kwang-Su"\},\{"family":"Yang","given":"See-Hun"\},\{"family":"Thomas","given":"Luc"\},\{"family":"Parkin","given":"Stuart S. P."\}],"issued":\{"date-parts":[["2014",5,23]]\}\}\}],"schema":"https://github.com/citation-style-language/schema/raw/master/csl-citation.json"\}$^3^ that is $\sim2.1$kOe when $M_{s}=520$ emu cm^-3^, $t=1$ nm, and $\Delta=2$ nm. From this, we find that $\frac{M_{s}^{2}}{2t}U\sim\frac{4\pi^{2}M_{s}^{2}wt\Delta}{t+\Delta}$ and $U\sim\frac{8\pi^{2}wt^{2}\Delta}{t+\Delta}\sim1.3\times{10}^{-22}$ m^3^, which agrees with the numerical calculations shown in Fig. S6b. On the other hand, $\frac{M_{s}^{2}}{2b^{2}t}V\sec\chi$ is the geometrical twisting induced magnetostatic interaction that has no dependence on twisting chirality. Hence, the eq. (S18) becomes

$$\mathcal{E}_{ms}\approx\frac{M_{s}^{2}}{2L}R\left( q \right)+\frac{M_{s}^{2}}{2t}\left[ U\cos^{2} \left( \psi-\chi\right)\sec\chi+\xi^{2}V \right]$$

(S19)

If eq. (S19) is plugged into the eqs. (S13), we finally obtain the eqs. of motion for chiral DWs on 3D twisted ribbons that include the magnetostatic energy as follows:

$$\dot{q}=\frac{\gamma\sec\chi}{1+\alpha^{2}}\left\{ -\frac{\pi\xi A_{ex}}{M_{s}}\cos\left( \psi+\chi\right)\pm\frac{\pi}{2}\Delta H_{p}\sin\left( \psi-\psi_{H} \right)\pm\frac{\pi}{2}\Delta H_{DM}\sin\left( \psi-\psi_{DM} \right)\mp\frac{\pi}{2}\alpha\Delta H_{SH}\cos\psi-\frac{\alpha M_{s}\Delta}{4wL}\frac{\partial R\left( q \right)}{\partial q}\mp\frac{M_{s}}{4wt}U\sin2\left( \psi-\chi\right) \right\}$$

$$\dot{\psi}=\frac{\gamma}{1+\alpha^{2}}\left\{ \pm\frac{\pi{\alpha\xi A}_{ex}}{M_{s}\Delta}\cos\left( \psi+\chi\right)-\frac{\pi}{2}\alpha\Delta H_{p}\sin\left( \psi-\psi_{H} \right)-\frac{\pi}{2}\alpha\Delta H_{DM}\sin\left( \psi-\psi_{DM} \right)-\frac{\pi}{2}\Delta H_{SH}\cos\psi-\frac{M_{s}}{4wL}\frac{\partial R\left( q \right)}{\partial q}\pm\frac{{\alpha M}_{s}}{4wt\Delta}U\sin2\left( \psi-\chi\right) \right\}$$

$$\dot{\chi}=\frac{-\frac{\sigma}{M_{s}\Delta}\tan\chi\pm\pi H_{DM}\sin\left( \phi-\chi\right)+\frac{\pi{\xi A}_{ex}}{2M_{s}\Delta}\cos\psi\sec\chi\mp\frac{M_{s}}{2wt\Delta}U\sin2\left( \psi-\chi\right)}{\frac{\pi^{2}\alpha}{6\gamma}\left[ \left( \frac{w}{\pi\Delta} \right)^{2}\sec^{2} \chi+\tan^{2} \chi\right]}.$$

(S17a,b,c)

Here

$$\sigma=\frac{2A_{ex}}{\Delta}+2K_{eff}\Delta+\frac{M_{s}^{2}}{2wt}U\cos^{2} \left( \psi-\chi\right)-\pi{\Delta M}_{s}H_{p}\cos\left( \psi-\psi_{H} \right)-\pi\Delta M_{s}H_{DM}\cos\left( \psi-\chi\right).$$

The eqs. (S17) agree well with those^7^ for current driven DWs having tilted angle in the flat strip wires but no geometrical twisting.


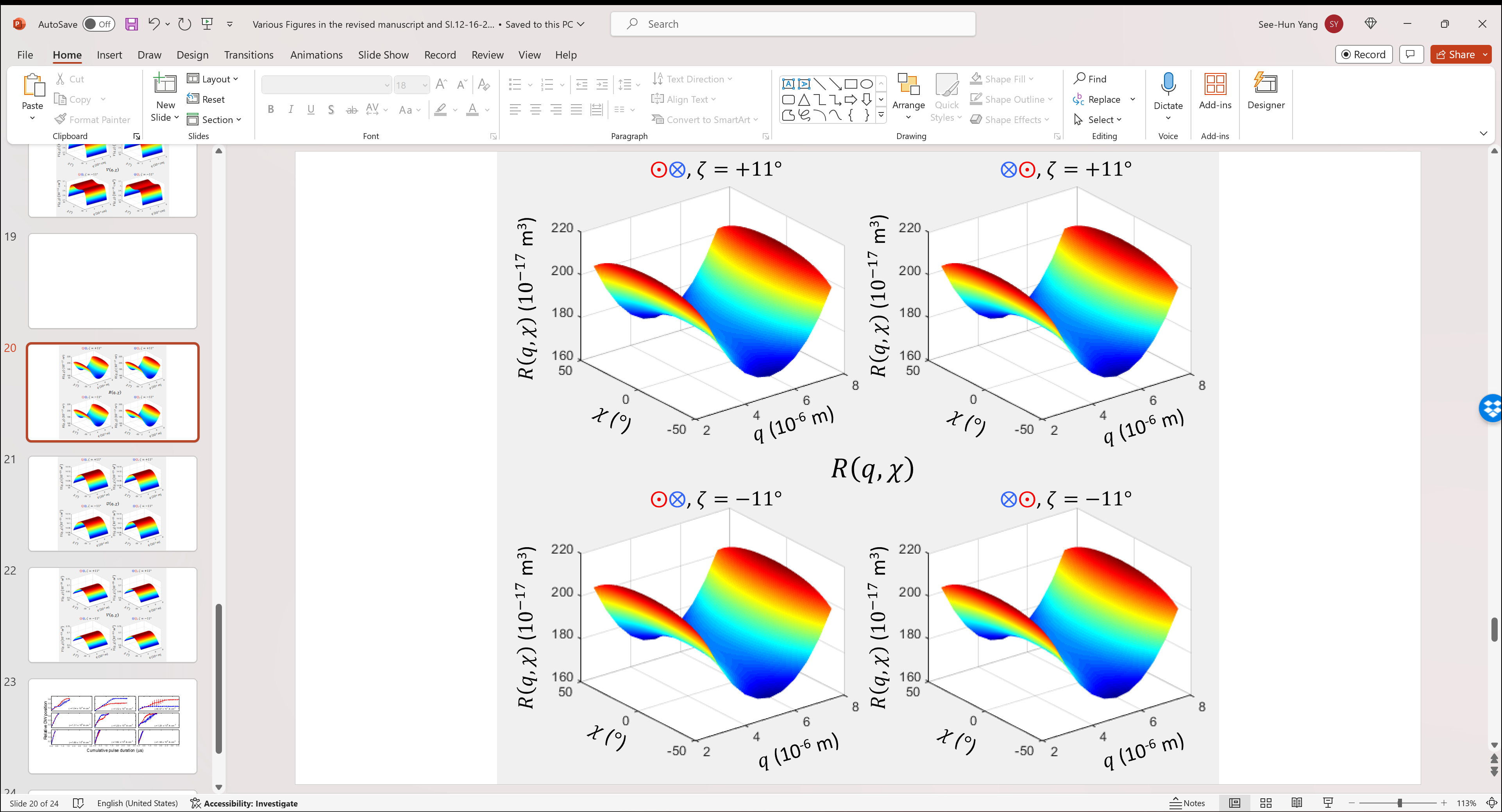


**aa**


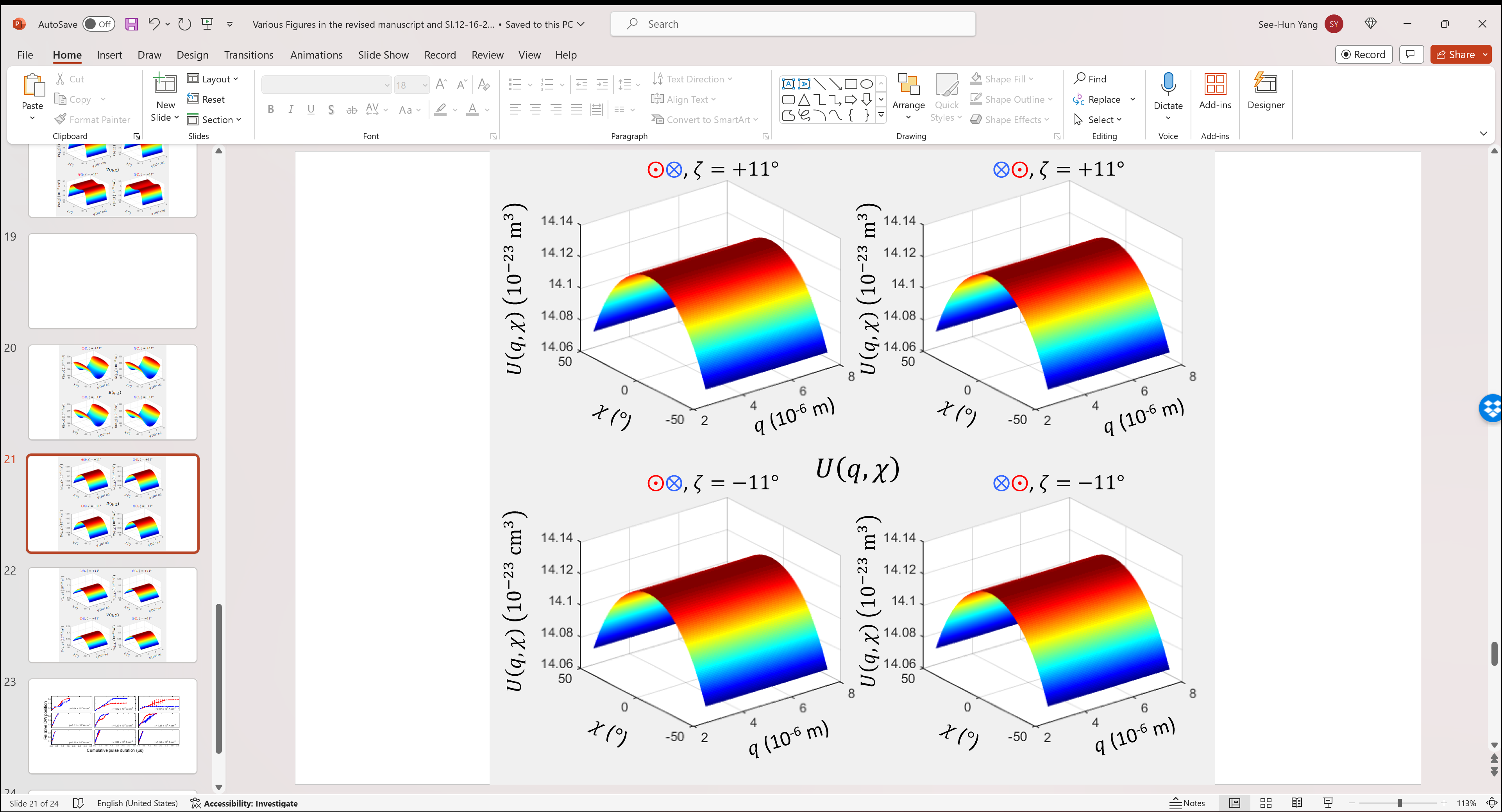

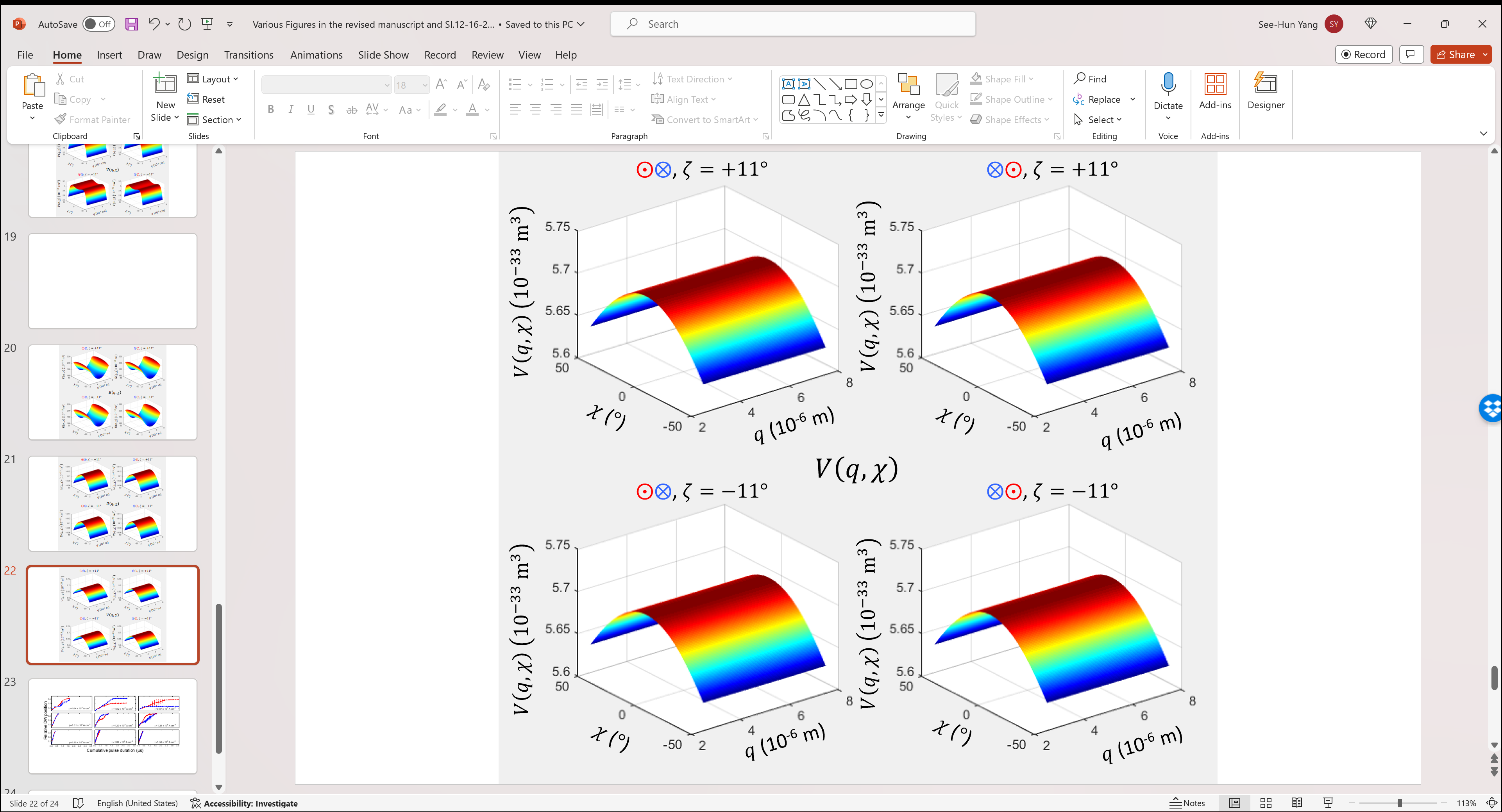


**ccba**

**bba**

**Fig. S6 | Numerically calculated parameters related to magnetostatic polar energy** $\boldsymbol{E}_{\boldsymbol{dip}}$**. a,** $R\left( q,\chi\right)$. **b,** $U\left( q,\chi\right)$, and **c,** $V\left( q,\chi\right)$. $t=1$ nm, $\Delta=2$ nm, $w=2.5$ μm, $L=10$ μm, and $\zeta=\pm11^{\circ}$ are used for the calculations. See the text.

**Supplementary Note 4: Additional device characterization**

SEM perspective views from devices used in this study are shown in Figs. S7 (various 3D twisted angles) and S8 (various ribbon widths). Some steps are observed on the surfaces of 3D twisted ribbons. Figure S5 shows the AFM results to measure the RMS roughness of the films over the suspended section of a device with ${+11}^{\circ}$ twist angle.

**c**

**b**

**a**


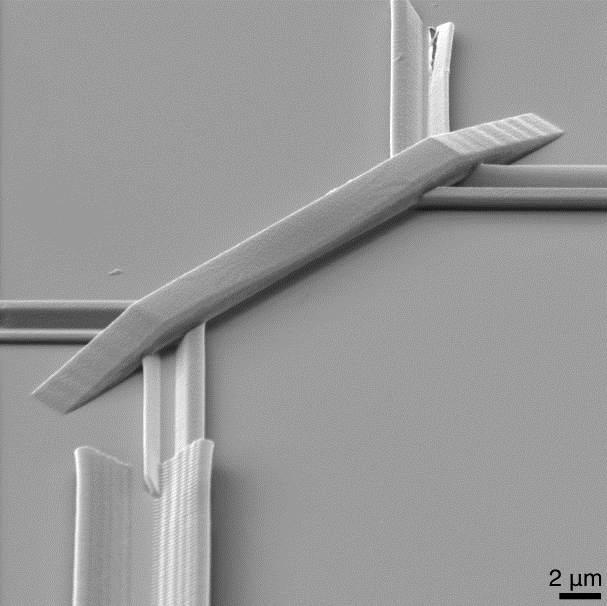

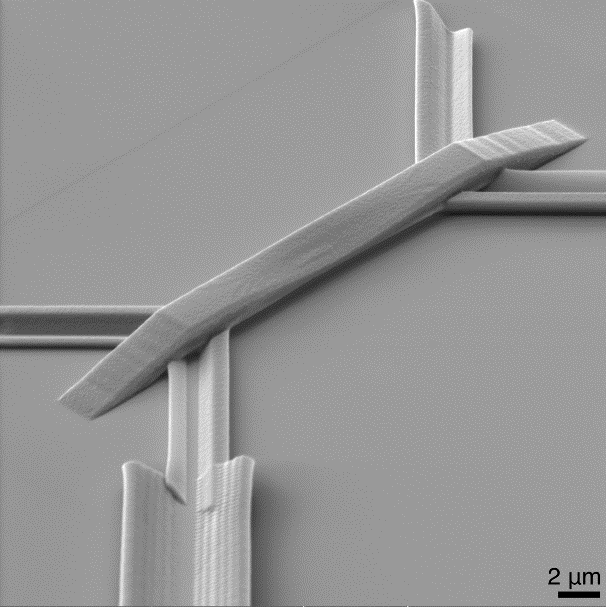

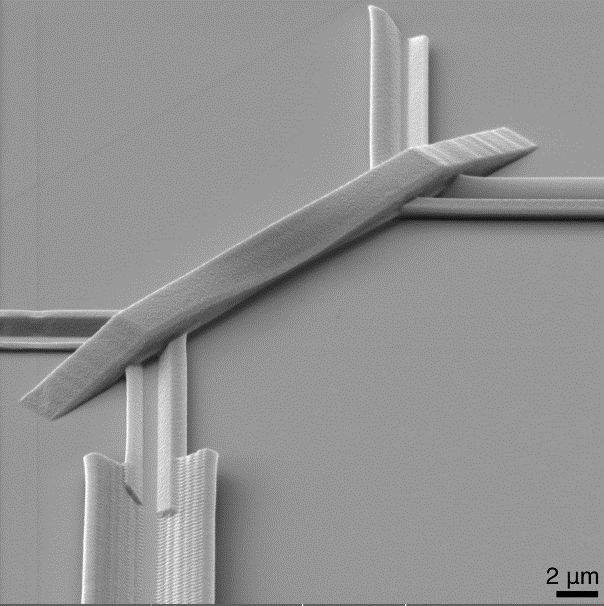


**Figure S7 |** SEM perspective image views from devices with $\zeta={+11}^{\circ}$ (**a**), ${+18}^{\circ}$ (**b**) and ${+26}^{\circ}$ (**c**) twisting angle and $w=2.5$ μm width.

**b**

**a**


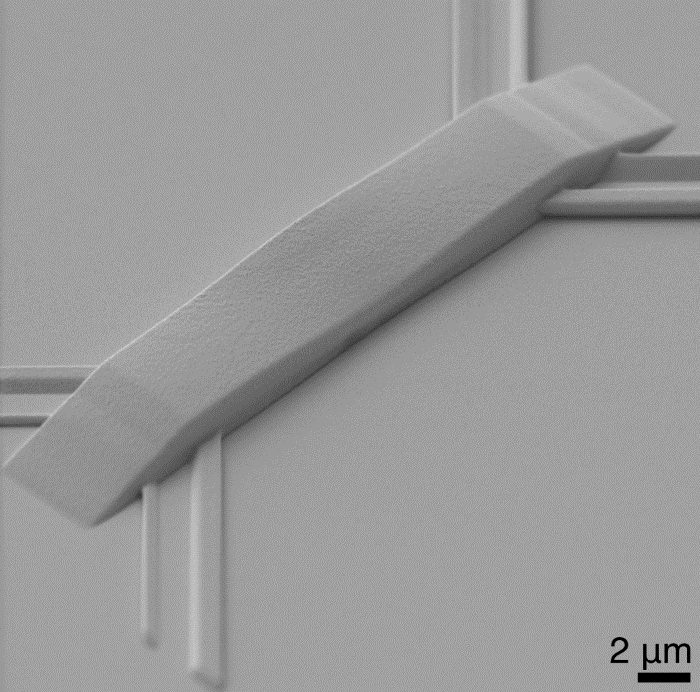

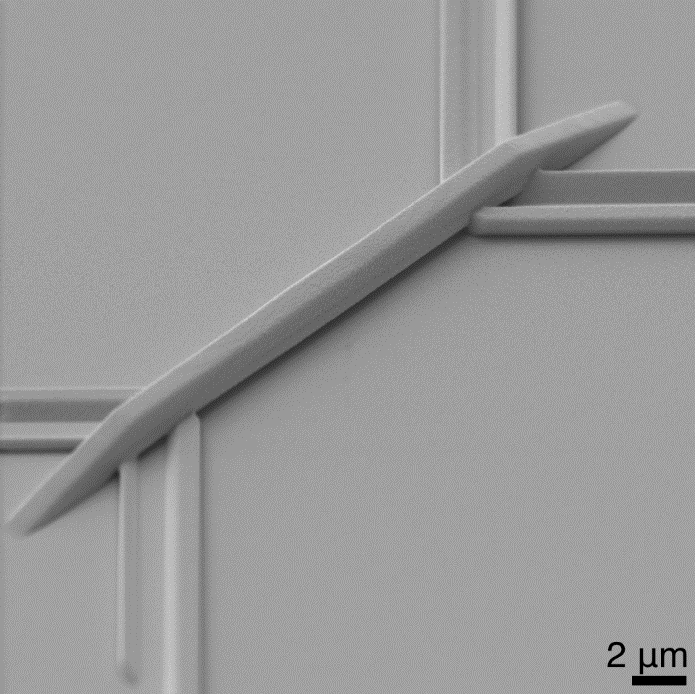


**Figure S8 |** SEM perspective image views from devices with $w=$1.25 μm (**a**) and 5 μm (**b**) and ${\zeta=+11}^{\circ}$.


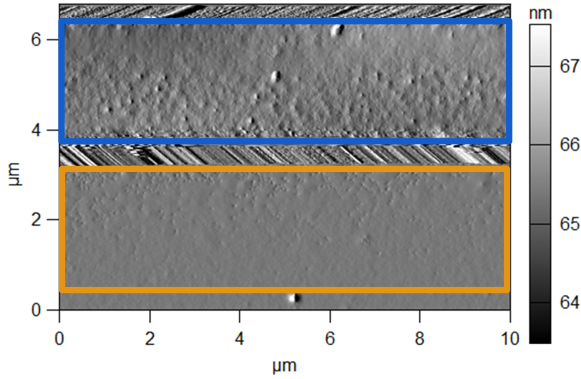


**Figure S9 |** AFM image scan on the surface of the device with ${\zeta=11}^{\circ}$ and $w=$2.5 μm (blue rectangle) and the surface of the contiguous glass substrate (orange rectangle). The regions bounded with colored rectangles are used to calculate the RMS roughnesses that are referred to in the main text: 0.37 nm for the device surface and 0.1 nm for the substrate.

**Supplementary Note 5: Hysteresis loops and micromagnetic parameters**

Magnetic properties of ferromagnetic (FM) and synthetic antiferromagnetic (SAF) films deposted on substrates are characterized by measuring magnetic hysteresis loops along easy (out-of-plane) and hard (in-plane) axes with vibrating sample magnetometry (VSM) as shown in Fig. S10 (FM) and S11 (SAF). For FM, we obtain the coercivity $H_{c}$ ~ 58 Oe, the saturation magnetization $M_{s}$ ~ 520 emu cm^-3^, and the effective anisotropy field $H_{eff}$ ~ 11 kOe that corresponds to the saturation field in the hard-axis hysteresis loop. As such, the effective anisotropy $K_{eff}=\frac{M_{s}H_{eff}}{2}=2.86\times{10}^{6}$ erg cm^-3^ and the crystalline anisotropy $K=K_{eff}+2\pi M_{s}^{2}\sim4.56\times{10}^{6}$ erg cm^-3^. As for the exchange constant, we estimate it to be $A_{ex}=34$ pJ m^-1^ based on the FM layer stacks 3 Co/7 Ni/3 Co and the reference^8^ by weighted averaging of reference values.

**b**

**a**

**Figure S10 |** Hysteresis loops of the FM film stack deposited on the substrate, measured by VSM along the hard axis (**a**) and the easy axis (**b**).

**b**

**a**


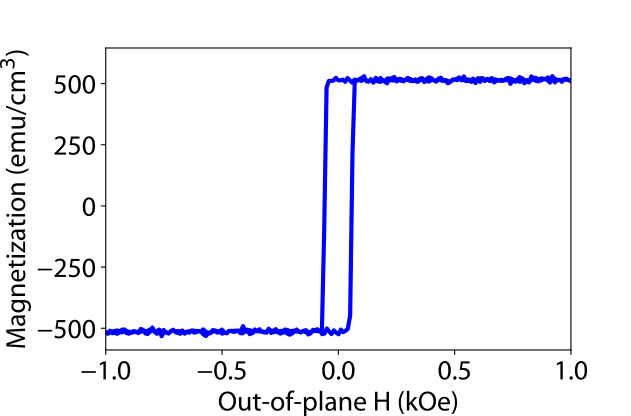

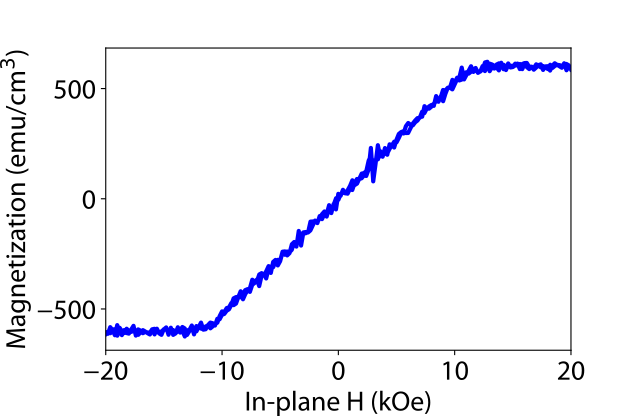

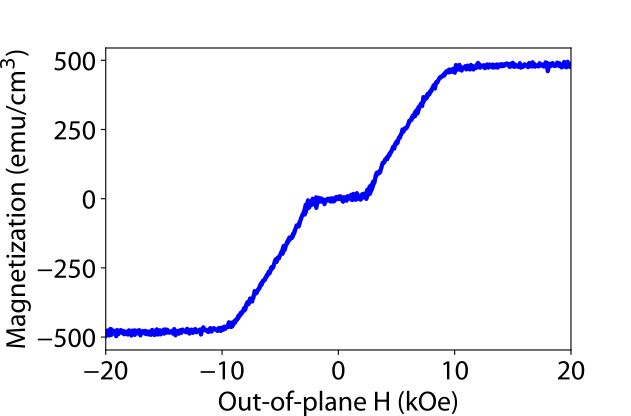

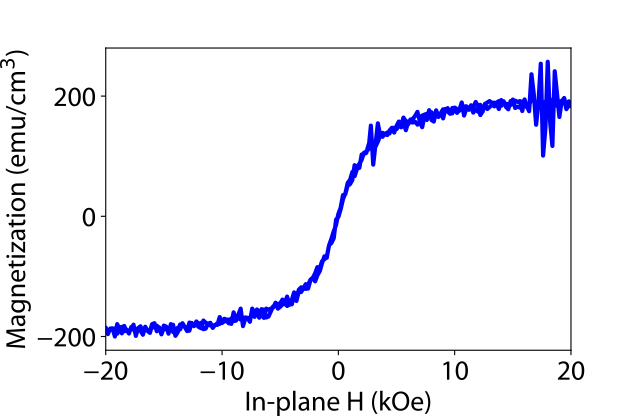


**Figure S11 |** Hysteresis loops of the SAF film stack deposited on the substrate, measured by VSM along the hard axis (**a**) and the easy axis (**b**).

From $K_{eff}$ and $A_{ex}$, we estimate the DW width parameter $\Delta=\sqrt{\frac{A_{ex}}{K_{eff}}}=10.1$ nm. Note here that the measurement of all key magnetic properties listed above on devices only is challenging except $H_{c}$ since the films on the contiguous substrate dominantly contribute to the VSM data. Kerr microscopy is used to measure hard-axis hysteresis loop on the device only from which $H_{c}$ is extracted. Flat and twisted regions are focused during the measurement (see Figs. S12 and S13). We find that $H_{c}$ ~ 170 Oe on the twisted regions that is larger than that for the entire substrate by nearly 3 times, as typically observed from the difference between small device and large blanket film. No significant difference between hysteresis curves of different $\zeta$’s from both the flat and the twisted regions is observed, thereby showing that the magnetic films for all samples are more or less similar to each other.

**b**

**a**

**d**

**c**


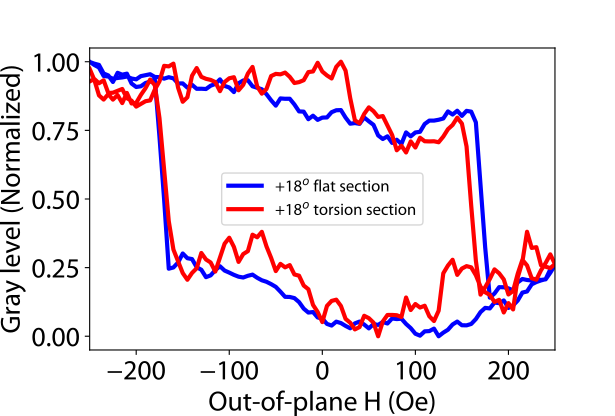

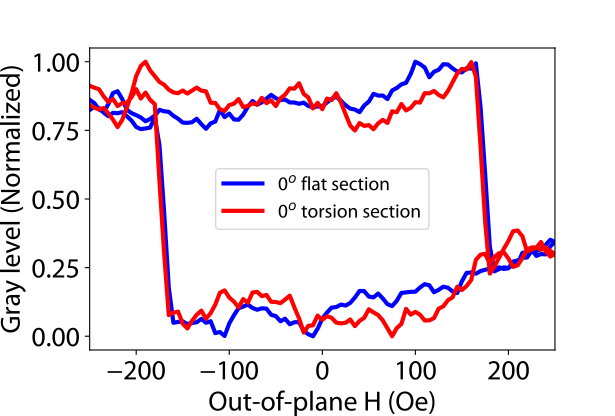

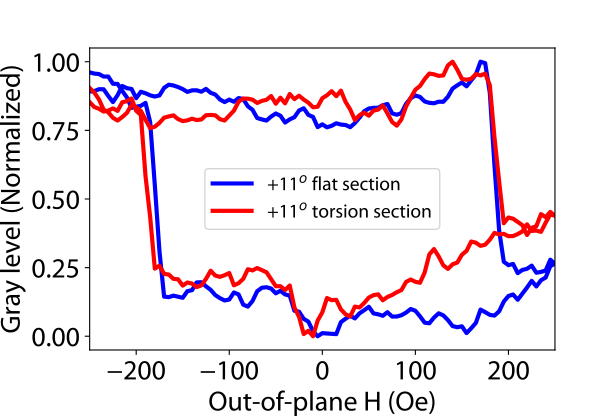

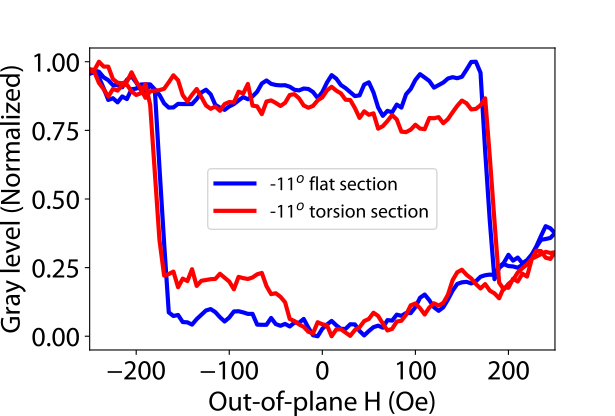


**Figure S12 |** Easy axis hysteresis loops for the FM stack over devices with $\zeta=0$ (**a**), +11 (**b**), -11 (**c**) and +18˚ (**d**) measured by Kerr microscopy. Blue and red solid curves correspond to the flat and the twisted regions of devices.


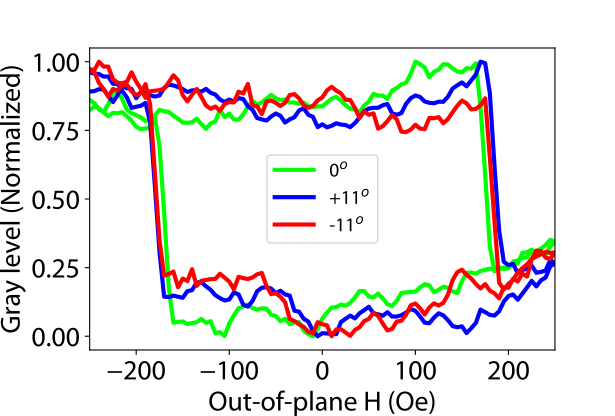


**Figure S13 |** Hysteresis loops for the ferromagnetic film stack over devices with 0, +11 and -11˚ twisting angles, measured at the point where the surface rotation is highest (the middle of the suspended section). Measured by magneto optic Kerr effect.

The effective interface induced DMI fields $H_{DM}\sim1$ kOe are extracted by fitting $v-H_{x}$ curves shown in Fig. 5a from which we have $D=M_{s}H_{DM}\Delta\sim0.52$ erg cm^-2^. If the volume spin-transfer torque is assumed to be small, the crossing point field $H_{cr}$ of the curve with $v-$axis corresponds to $-H_{DM}$, i.e. $H_{cr}\sim-H_{DM} ADDIN ZOTERO\_ITEM CSL\_CITATION \{"citationID":"myABqMCJ","properties":\{"formattedCitation":"\backslash\backslash super 3,9\backslash\backslash nosupersub\{\}","plainCitation":"3,9","noteIndex":0\},"citationItems":[\{"id":20,"uris":["http://zotero.org/users/local/pl8qgtBf/items/D8AB8MM9"],"itemData":\{"id":20,"type":"article-journal","abstract":"Spin-polarized currents provide a powerful means of manipulating the magnetization of nanodevices, and give rise to spin transfer torques that can drive magnetic domain walls along nanowires. In ultrathin magnetic wires, domain walls are found to move in the opposite direction to that expected from bulk spin transfer torques, and also at much higher speeds. Here we show that this is due to two intertwined phenomena, both derived from spin-orbit interactions. By measuring the influence of magnetic fields on current-driven domain-wall motion in perpendicularly magnetized Co/Ni/Co trilayers, we find an internal effective magnetic field acting on each domain wall, the direction of which alternates between successive domain walls. This chiral effective field arises from a Dzyaloshinskii-Moriya interaction at the Co/Pt interfaces and, in concert with spin Hall currents, drives the domain walls in lock-step along the nanowire. Elucidating the mechanism for the manipulation of domain walls in ultrathin magnetic films will enable the development of new families of spintronic devices.","container-title":"Nature Nanotechnology","DOI":"10.1038/nnano.2013.102","ISSN":"1748-3395","issue":"7","language":"en","license":"2013 Nature Publishing Group","page":"527-533","source":"www.nature.com","title":"Chiral spin torque at magnetic domain walls","volume":"8","author":[\{"family":"Ryu","given":"Kwang-Su"\},\{"family":"Thomas","given":"Luc"\},\{"family":"Yang","given":"See-Hun"\},\{"family":"Parkin","given":"Stuart"\}],"issued":\{"date-parts":[["2013",7]]\}\}\},\{"id":480,"uris":["http://zotero.org/users/local/pl8qgtBf/items/B29KERZD"],"itemData":\{"id":480,"type":"article-journal","abstract":"Domain walls can be driven by current at very high speeds in nanowires formed from ultra-thin, perpendicularly magnetized cobalt layers and cobalt/nickel multilayers deposited on platinum underlayers due to a chiral spin torque. An important feature of this torque is a magnetic chiral exchange field that each domain wall senses and that can be measured by the applied magnetic field amplitude along the nanowire where the domain walls stop moving irrespective of the magnitude of the current. Here we show that this torque is manifested when the magnetic layer is interfaced with metals that display a large proximity-induced magnetization, including iridium, palladium and platinum but not gold. A correlation between the strength of the chiral spin torque and the proximity-induced magnetic moment is demonstrated by interface engineering using atomically thin dusting layers. High domain velocities are found where there are large proximity-induced magnetizations in the interfaced metal layers.","container-title":"Nature Communications","DOI":"10.1038/ncomms4910","ISSN":"2041-1723","language":"en","license":"2014 Nature Publishing Group","page":"3910","source":"www.nature.com","title":"Chiral spin torque arising from proximity-induced magnetization","volume":"5","author":[\{"family":"Ryu","given":"Kwang-Su"\},\{"family":"Yang","given":"See-Hun"\},\{"family":"Thomas","given":"Luc"\},\{"family":"Parkin","given":"Stuart S. P."\}],"issued":\{"date-parts":[["2014",5,23]]\}\}\}],"schema":"https://github.com/citation-style-language/schema/raw/master/csl-citation.json"\}$^3,9^. Due to a large uncertainty in the cross point caused by DW pinning, we fitted $v-H_{x}$ curves and estimated $H_{DM}$ to be ~1 kOe. Consequently, we have $D=M_{s}H_{DM}\Delta\sim0.52$ erg cm^-2^.

**Supplementary Notes 6: Twisted angle longitudinal profiles**

Note that our 3D twisted ribbons have two opposite chiralities along the length direction: the ribbon over one half from one end to the halfway is twisted in one direction while over the other half the ribbon is twisted in the opposite direction. To show this quantitatively, the designed twisted angle longitudinal profiles are shown in Fig. S14. The effective twisting length $L$, corresponds to the section of the ribbon over which the twist parameter, i.e. the derivative of the curve in Figure S14, is constant. Since each ribbon has two halves with opposite twist angles, there are two sections with an approximately constant twist parameter, e.g. ~ [0.15;0.35] and [0.65;0.85] in figure S14, both with the same effective twisting length $L$.

Twist angle profile over the twisted section


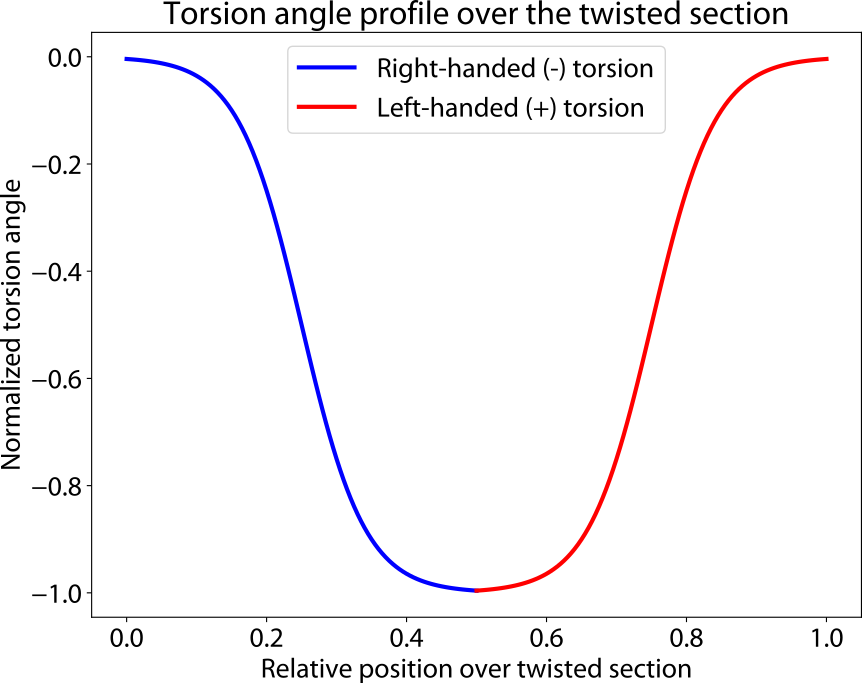


Relative position over twisted section

Normalized Twist angle

**Figure S14 |** Normalized twist angle profiles with $\zeta=-11^{\circ}$ for the first half and $=11^{\circ}$ for the other half, over the normalized ribbon length.

**Supplementary References**

1. Gaididei, Y. *et al.* Magnetization in narrow ribbons: curvature effects. *J. Phys. A: Math. Theor.* **50**, 385401 (2017).

2. Gaididei, Y., Kravchuk, V. P. & Sheka, D. D. Curvature Effects in Thin Magnetic Shells. *Phys. Rev. Lett.* **112**, 257203 (2014).

3. Ryu, K.-S., Yang, S.-H., Thomas, L. & Parkin, S. S. P. Chiral spin torque arising from proximity-induced magnetization. *Nat. Commun.* **5**, 3910 (2014).

4. Garg, C., Yang, S.-H., Phung, T., Pushp, A. & Parkin, S. S. P. Dramatic influence of curvature of nanowire on chiral domain wall velocity. *Sci. Adv.* **3**, e1602804 (2017).

5. Volkov, O. M. *et al.* Chirality coupling in topological magnetic textures with multiple magnetochiral parameters. *Nat. Commun.* **14**, 1491 (2023).

6. Sheka, D. D. *et al.* Nonlocal chiral symmetry breaking in curvilinear magnetic shells. *Commun. Phys.* **3**, 1–7 (2020).

7. Boulle, O. *et al.* Domain Wall Tilting in the Presence of the Dzyaloshinskii-Moriya Interaction in Out-of-Plane Magnetized Magnetic Nanotracks. *Phys. Rev. Lett.* **111**, 217203 (2013).

8. Kuz’min, M. D., Skokov, K. P., Diop, L. V. B., Radulov, I. A. & Gutfleisch, O. Exchange stiffness of ferromagnets. *Eur. Phys. J. Plus* **135**, 301 (2020).

9. Ryu, K.-S., Thomas, L., Yang, S.-H. & Parkin, S. Chiral spin torque at magnetic domain walls. *Nat. Nanotechnol.* **8**, 527–533 (2013).
